# Atlas of human dental pulp cells at multiple spatial and temporal levels based on single-cell sequencing analysis

**DOI:** 10.3389/fphys.2022.993478

**Published:** 2022-10-04

**Authors:** Huihui Ren, Quan Wen, Qingxuan Zhao, Nan Wang, Yuming Zhao

**Affiliations:** ^1^ Department of Pediatric Dentistry, Peking University School and Hospital of Stomatology and National Center of Stomatology and National Clinical Research Center for Oral Diseases and National Engineering Laboratory for Digital and Material Technology of Stomatology and Beijing Key Laboratory of Digital Stomatology and Research Center of Engineering and Technology for Computerized Dentistry Ministry of Health and NMPK Key Laboratory for Dental Materials, Beijing, China; ^2^ First Clinical Division, Peking University School and Hospital of Stomatology, Beijing, China

**Keywords:** tooth development, single-cell RNA sequencing, odontoblasts, dental stem cells, pulp regeneration

## Abstract

The dental pulp plays a crucial role in the long-term maintenance of tooth function. The progress of endodontic treatment and pulp tissue regeneration engineering has made pulp-regeneration therapy promising in clinical practice. However, the mechanisms of pulp regeneration and the role of dental stem cells in development and regeneration have not been fully elucidated. Bridging the gaps between clinical operation and basic research is urgently needed. With the application of single-cell sequencing technology in dental research, the landscapes of human dental pulp cells have begun being outlined. However, the specific cellular heterogeneity of dental pulp cells, especially that of dental stem cells, at different spatial and temporal levels, is still unclear. In this study, we used single-cell RNA sequencing analysis of pulp samples at four different developmental stages and combined the findings with immunohistochemical staining to explore the development of dental pulp and stem cells. The results revealed temporal changes in the proportion of pulp cells during development. For example, mononuclear phagocytes accounted for a higher proportion in early samples. Odontoblasts identified by *DMP1* had a higher expression of ion channel-related and neurodevelopment-related genes. Subpopulations were identified in fibroblasts, odontoblasts, and mesenchymal stem cells. We identified a subclass of odontoblasts that expresses *DGKI* and *RRBP1* present in early developmental samples. A population of earlier mesenchymal stem cells expressed the *SEPTIN* gene, which may have greater proliferative and differentiation potential. Furthermore, dental pulp stem cells can differentiate into two directions: mineralization and myogenesis. In summary, the specific cellular heterogeneity of dental pulp cells was revealed at different spatial and temporal levels. These findings may shed light on the mechanism of tooth development. The gene expression profile of developing pulp cells may help to select cells for regenerative engineering and improve the success of dental pulp regeneration.

## Introduction

Dental pulp plays a fundamental role in tooth formation, resistance to infection or trauma, and dentin restoration. Previous studies have established a rough general framework describing pulp development, histopathology, and function (Rombouts et al., 2017). Nevertheless, there are still no systematic and consistent conclusions regarding the cellular heterogeneity and gene profile of pulp cells. Pulp revascularization and pulp regeneration are now being used in clinical practice based on research in pulp tissue engineering to recreate vital pulp cells (Eramo et al., 2018; Shabahang, 2013; Xuan et al., 2018). Seed cells, the key to pulp tissue engineering, are derived from various dental stem cells containing human dental pulp stem cells (DPSC), stem cells from the root apical papilla (SCAP), and stem cells from human exfoliated deciduous teeth (SHED) (Gronthos et al., 2000; Miura et al., 2003; Sonoyama et al., 2006). These stem cells are named after their original source and express an immunophenotype similar to human bone marrow stromal cells (BMSC) *in vitro* rather than hematopoietic stem cells, although they are localized in the microvasculature *in vivo* (Pagella et al., 2021a; Shi and Gronthos, 2003). Some studies have identified heterogeneous populations of dental pulp stem cells using surface markers such as CD146 and CD90 (Matsui et al., 2018; Yasui et al., 2016) and demonstrated their improved proliferative capacity and differentiation potential. However, the status and role of human dental stem cells *in vivo* have not yet been fully characterized in current studies. The specific regulatory mechanism of pulp development and the heterogeneity of stem cells remain to be clarified.

Recently, several studies have introduced single-cell RNA sequencing into dental research and have developed the composition of human dental pulp cells in terms of gene expression (Caetano et al., 2021; Krivanek et al., 2020; Pagella et al., 2021b). The genes to identify different types of dental cells have been identified by using bioinformatics analysis (Krivanek et al., 2020; Pagella et al., 2021b), but it is challenging to decipher similarities and differences between cell populations in various spatial and temporal stages, as well as the role of intercellular communication.

In this study, we dissected information specific to dental pulp cells using the results of single-cell RNA sequencing of the samples from four teeth, one of which was collected from our dental clinic and the remaining three were samples extracted from the Gene Expression Omnibus (GEO) database. We compared similarities and differences in pulp cell composition, number, function, and cellular communication in different samples. Furthermore, we performed a detailed analysis and interpretation of fibroblasts, odontoblasts, and mesenchymal stem cell (MSC) compositions, and identified intracellular variability within these cell populations. Developing odontoblasts, potential primitive stem cells, and actively differentiated stem cell populations were identified. The analysis of odontoblasts and stem cell transcription factors also provides information on potential developmental regulatory mechanisms, which will help to further explore pulp regeneration in the future.

## Materials and methods

### Sample collection and database source

The human teeth samples used in our study were extracted for orthodontic or other clinical needs at the Emergency Department of Peking University Hospital of Stomatology in 2020 and 2021. We collected mature and immature teeth. This study was approved by the Biomedical Ethics Committee of Peking University School of Stomatology (PKUSSIRB-202060197). We collected an immature third molar in the stage of early root development (shorter than one-third of the root completed) from a 13-year-old child for single-cell RNA sequencing, which was designated as Young pulp 1 (Y1). Sequencing data were uploaded to the Gene Expression Omnibus (GEO) database as GSE202476. We also extracted raw data from three samples described in previous studies (Krivanek et al., 2020; Pagella et al., 2021b) and GEO datasets (GSM4365609, GSM4365610, and GSM4998458). One sample (GSM4365609) from the apical papilla of the tooth with two-thirds of the completed root was designated Young pulp 2 (Y2). The other two samples (GSM4365610 and GSM4998458) obtained from the pulp of the mature teeth were designated Adult pulp 1 and 2 (A1 and A2). Detailed information is provided in Supplementary Table S1.

### Single-cell suspension preparation

An immature pre-eruptive tooth was extracted from a 13-year-old boy and immediately transported to the laboratory in 4°C in a Minimum Essential Medium α (α-MEM, Gibco, United States) with 10% penicillin–streptomycin (Solarbio, China) solution immediately. Soft dental pulp tissue was harvested and washed three times in phosphate-buffered saline (PBS, Gibco, United States) in a super clean bench. Subsequently, the pulp tissue was shred into small pieces (<1 mm^3^) using ophthalmic scissors and centrifuged at 4°C, 1,200 rpm for 5 min. The precipitation was digested for 40 min in 3 mg/ml Type 1 collagenase (Sigma-Aldrich, United States) at 37°C, and agitated at 300 rpm in an oscillation box, and pipetted 8–10 times every 8–10 min. The digestion was stopped with 2% fatal bovine serum (FBS, ABW, Uruguay), and then, the solution was filtered through a 40-μm cell strainer. The filtrate was centrifuged at 4°C, 1,350 rpm, and 10 min three times, and the supernatant was removed. The pellet was then resuspended in PBS including 2% FBS and filtered through a 40-μm cell strainer.

### Single-cell RNA sequencing

To remove red blood cells and improve cell activity, we used 1 × red blood cell lysis solution at 4°C to remove blood cells. We used chromium single-cell 3ʹ reagent kits v3 to construct the cDNA library according to the manufacturer’s protocols, and libraries were sequenced on the Illumina Nova seq 6,000 system in the PE150Nova mode. The quantity used for sequencing was about 50 k reads per cell. Cell quality control, detection, reference genome comparison, and cell-gene expression matrix generation were completed using Cell Ranger 4.0.0, the official 10x Genomics analysis software. All sequencing work was conducted in cooperation with Beijing MicroRead Genetics Co., Ltd. (Beijing, China).

### Quality control and analysis

Expression matrix files for subsequent analyses were generated from gene counts and unique molecular identifier (UMI) counts. Any batch effects between our sequencing sample data and data from the public database were removed by Harmony (Korsunsky et al., 2019).

Cells were screened by gene count in the range of 300 and 7,000 and UMI count under 30,000. Cells containing more than 10% mitochondrial content were excluded. We used Seurat v2.3 (Satija et al., 2015) for dimension-reduction and clustering. Gene expressions were normalized and scaled using NormalizeData and ScaleData. The top 2,000 variable genes were selected by FindVariableFeatures for principal component analysis (PCA). Cells were grouped into clusters by FindClusters using the first 20 principal components and a resolution parameter of 1.0. For subclustering of various cell types, we set the resolution to 1.2. The Uniform Manifold Approximation and Projection (UMAP) algorithm was applied to perform two-dimensional visualization of cells. Seurat FindMarkers selected genes as differentially expressed genes (DEGs) that were expressed in more than 10% of the cells in a cluster with a mean fold change greater than 0.25 based on the Wilcox likelihood ratio test and default parameters. The cell-type status of each group was determined on the basis of the expression of typical markers found in DEGs, together with knowledge of the relevant literature. All data analysis work was performed in cooperation with Singleron Biotechnologies (Nanjing, China).

### Pathway enrichment analysis

To investigate the potential functions of different cell types, an analysis of Gene Ontology (GO) and Kyoto Encyclopedia of Genes and Genomes (KEGG) was performed using the “clusterProfiler” R package v4.0.2 (Yu et al., 2012). Pathways with a value of p_adj less than 0.05 were deemed to be significantly enriched. For the gene set variation analysis (GSVA) and the pathway enrichment analysis, the mean gene expression of each cell type was included as input data using the GSVA package v1.34.0 (Hänzelmann et al., 2013).

### Trajectory analysis

Pseudotime trajectory analysis was performed with Monocle2 (Qiu et al., 2017). To construct the trajectory, the top 2,000 highly variable genes were selected by using Seurat v2.3 FindVariableFeatures, and the dimensionality reduction was performed by DDRTree. The trajectory was visualized by plot_cell_trajectory.

### Cellphone DB

Cell–cell interaction was estimated using Cellphone DB (Efremova et al., 2020) based on known ligand–receptor pairs. The number of reciprocals used to calculate the null distribution of the average expression of the ligand–receptor pairs in random cell identities was set to 1,000. Thresholds for individual ligands or receptor expressions were determined based on the average log gene expression distribution of all genes in each cell type. The predicted interaction pairs with *p*-value < 0.05 and mean log expression > 0.1 were considered significant and were visualized by using circlize (0.4.10) R package.

### Single-cell regulatory network inference and clustering analysis

We used pyscenic (version 0.11.0) to perform a single-cell regulatory network analysis. The analysis was conducted according to the protocol described in single-cell regulatory network inference and clustering (SCENIC) working process (Van De Sande et al., 2020). The “pyscenic grn” function was first used to generate regulatory networks of co-expression genes using the “grnboost2” method. AUCell analysis was also performed using the “pyscenic aucell” function with parameters “rank_threshold” 5000, “auc_threshold” 0.05, and “nes_threshold” 3.

### Single-cell entropy analysis

Single-cell lineage inference using cell expression similarity and entropy (SLICE) (version 0.99.0) (Guo et al., 2017) was used to assess cell stemness by gene expression entropy based on single-cell expression profiles. After removing external RNA controls consortium (ERCC) spike-ins and ribosomal genes, a SLICE object was created to perform bootstrap calculation of single-cell gene entropy values by using the getEntropy function.

### Sample preparation for histological evaluation

For histological evaluation, the molars or the dental pulp of the molars were fixed in 4% paraformaldehyde for 24 h. For some samples, we directly took out the pulp tissue for sectioning. Teeth with crown were decalcified in a 17% ethylene diamine tetraacetic acid (EDTA) solution for 1 year at 37°C. All samples were then dehydrated in graded ethanol, embedded in paraffin, and cut into 5-μm-thick sections.

### Immunohistochemistry

Paraffin sections were dewaxed with a series of ethanol solutions (100%–70%) and washed again with distilled water and PBS. Antigen retrieval was conducted by heating at 70°C in an EDTA buffer pH 9 for 20 min. After cooling to room temperature, the sections were washed in a PBS solution three times and incubated in 3% hydrogen peroxide for 20 min to remove endogenous peroxidase. Next, 10% goat serum (ZSGB-BIO, China) was used for 20 min to avoid any non-specific reaction. The primary antibodies were incubated at 4°C overnight. Specimens were incubated with biotin-conjugated IgG and horseradish peroxidase-conjugated streptavidin (ZSGB-BIO, China) for 20 min, respectively. The sections were then visualized using 3, 3′-diaminobenzidine-tetrahydrochloride (ZSGB-BIO, China) and washed in distilled water. Finally, hematoxylin staining was added for nuclear staining. All sections were observed under a light microscope (Olympus, Japan).

The following antibodies were used in our study: CD163 (1:500, Abcam, United States), SLC12A2 (1:100, Proteintech, China), ST8SIA1 (1:100, Proteintech), CD24 (1: 50, Santa Cruz, United States), WISP1 (1:100, Proteintech), CD146/MCAM (1: 250, Abcam), and CD90 (1:200, Abcam).

## Results

### Landscapes of multiple human dental pulps

**FIGURE 1:**
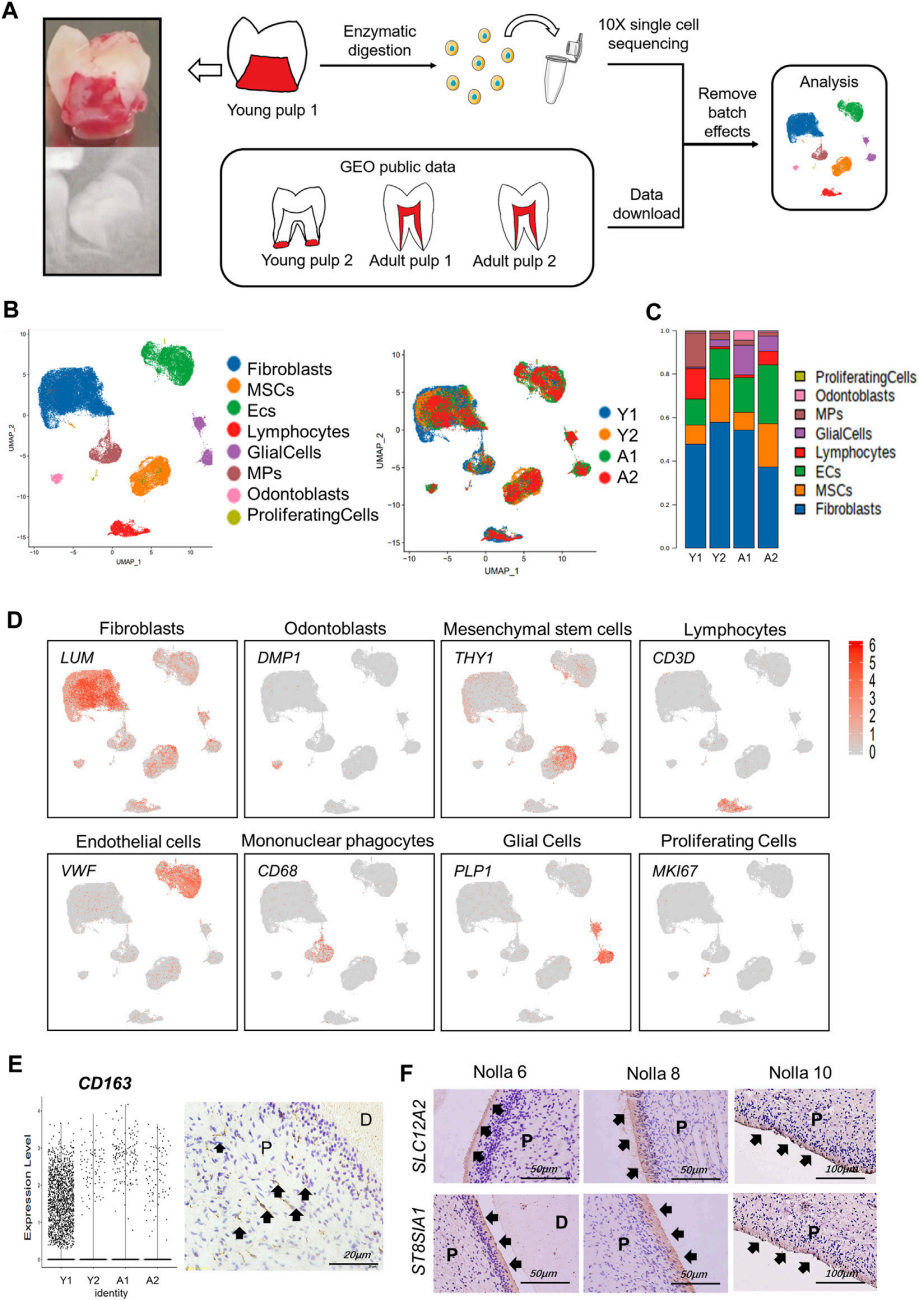
Gene expression landscape of multiple human dental pulps. **(A)** Study overview. (Left) Sagittal section view and radiograph of the collected tooth samples. **(B)** Human dental pulp cell types and the distributions of the four samples visualized in UMAP plots. MSCs: mesenchymal stem cells. ECs: endothelial cells. MPs: mononuclear phagocytes. **(C)** Relative proportion of eight cell types in the four samples. **(D)** Feature plots of unique marker genes in various cell clusters. **(E)** Vlnplot of *CD163* in the four samples (left) and immunohistochemical staining in the crown of young pulp (right, black arrows point to active signals). **(F)** Immunohistochemical staining of SCL12A2 and ST8SIA1 (black arrows) in odontoblasts at Nolla stages 6, 8, and 10. P, pulp; D, dentin.

Y1 was sequenced using the 10X Genomics chromium platform, from which 12,620 cells were obtained. Y2 of GSM4365609 was in a later stage of development than Y1, and only the apical papilla of the root was extracted, which participates in the elongation and maturation of the root (Driesen et al., 2021). In addition, two samples from mature tooth pulp (A1 and A2), for a total of four samples, were analyzed in our study to explore the spatial and temporal characterization of the gene expression in human tooth development (Figure 1A; Supplementary Table S1). After removing batch effects and homogenization, we used SingleR and referenced previous studies (Chiba et al., 2020; Krivanek et al., 2020; Pagella et al., 2021b) to group all pulp cells into eight different types (Figures 1A,B; Supplementary Table S2). *COL1A1* and *LUM* were used to define fibroblasts, *DMP1* to odontoblasts, and MSCs were defined by *THY1/CD90* and *ACTA2* (Figure 1D). *CD68* and *VWF* were used to mark mononuclear phagocytes (MPs) and endothelial cells (ECs), respectively. In terms of cell types, the four samples did not differ from each other (Supplementary Figure S1A). However, the proportion of each cell type was different (Figure 1C; Supplementary Table S3). We harvested 46,428 cells in total. Fibroblasts and ECs were always the main cell populations in each sample. It was interesting that Y1 had more MPs and lymphocytes, even more than MSCs. As a marker gene for MPs, *CD163* was significantly expressed in Y1, while immunohistochemical staining showed a positive expression of CD163 in the crown of young pulp (Figure 1E). When the MPs were further characterized (Supplementary Table S2), conventional dendritic cells (cDCs) were found to be significantly more predominant in Y1, while monocytes were present in large numbers in A2 (Supplementary Figure S1B). Differences in cell proportion also existed between two mature samples A1 and A2: A1 was characterized by more ECs and glial cells, while A2 had more MSCs and ECs.

As a special cell type in the pulp, odontoblasts account for a small portion. Because the characteristics and gene expression profiles of odontoblasts and their fate determination are not well-understood, an in-depth investigation of human odontoblasts was performed. We found that genes that encoded ion channel proteins and ion-binding proteins were highly expressed, such as *TRPM7*, *SLC12A2*, and *SPOCK3*. Neurodevelopment-related genes, such as *ST8SIA1* and *MAP1B*, were also significantly expressed in odontoblasts (Table 1; Supplementary Figure S1C). Immunohistochemical results demonstrated that *SLC12A2* and *ST8SIA1* were expressed in odontoblasts in the temporal stages of development (Figure 1F). Other genes encoded secreted and membrane-associated proteins in odontoblasts (Table 1).

**TABLE 1:** The top 20 genes in odontoblasts.

Gene_id	p_val	avg_logFC	pct.1	pct.2	p_val_adj
TRPM7	0	2.647467	0.853	0.218	0
SLC12A2	0	2.352713	0.755	0.17	0
ST8SIA1	0	2.209194	0.602	0.015	0
MAP1B	0	2.072654	0.878	0.373	0
SPOCK3	0	2.023397	0.523	0.006	0
SEMA3E	0	2.004852	0.531	0.038	0
CLU	0	1.802231	0.969	0.738	0
PTPRK	0	1.789348	0.548	0.101	0
GNAI1	0	1.763201	0.578	0.13	0
DTNBP1	0	1.722465	0.657	0.19	0
PHEX	0	1.716469	0.385	0.002	0
MMP20	0	1.704506	0.375	0.001	0
NES	0	1.688049	0.91	0.5	0
WISP1	0	1.672285	0.39	0.01	0
FEZ2	9.10E-201	1.633294	0.654	0.287	2.40E-196
DMP1	0	1.558536	0.315	0.001	0
COL27A1	0	1.553733	0.443	0.068	0
SORBS2	1.31E-249	1.541778	0.641	0.22	3.45E-245
SCIN	9.11E-306	1.524694	0.587	0.149	2.40E-301
GPC3	2.29E-251	1.459874	0.614	0.196	6.04E-247

### Transcriptional differences and intercellular communications of human dental pulp cells

**FIGURE 2:**
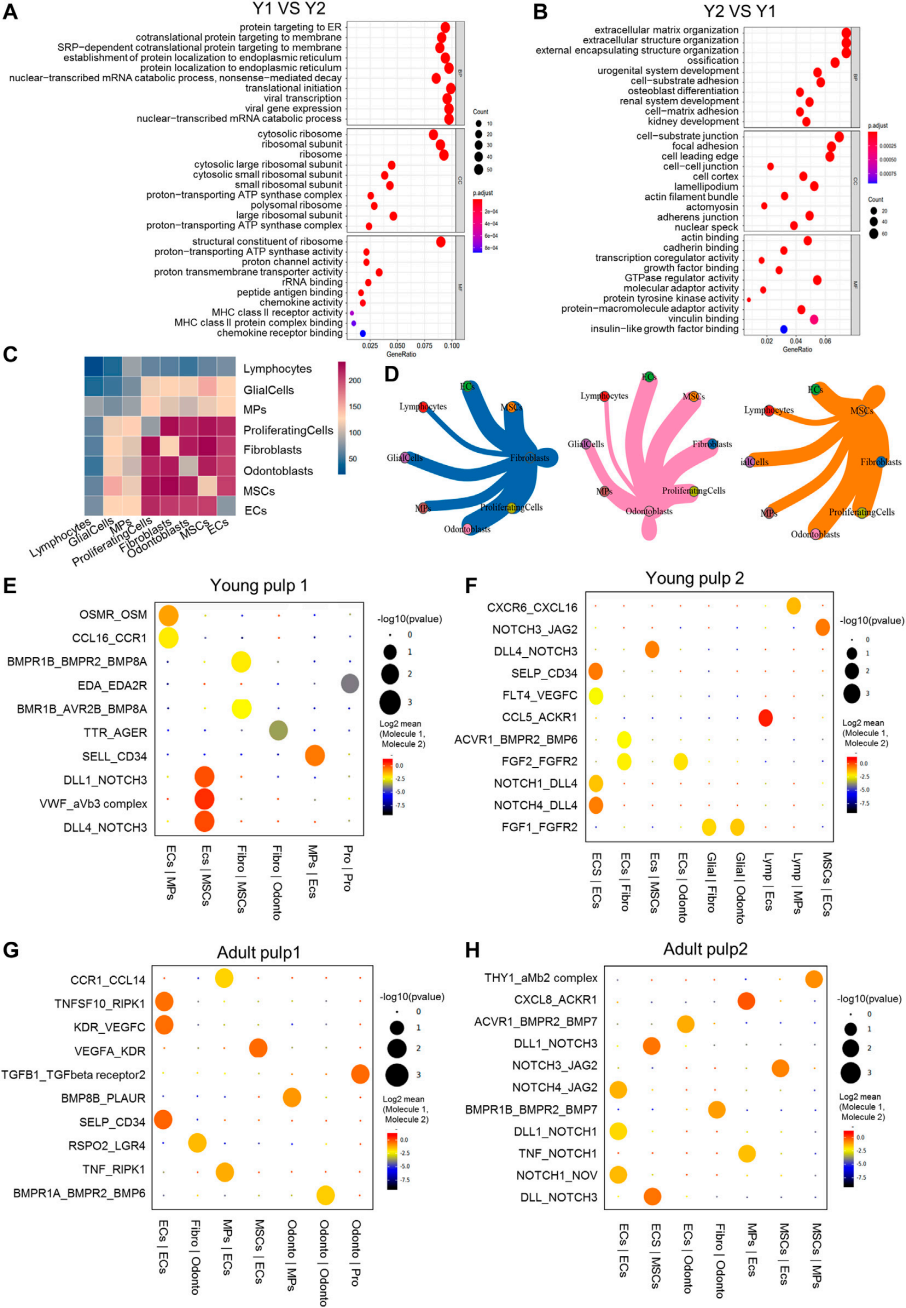
Transcriptional differences and intercellular communications of human dental pulp cells. **(A)** GO enrichment analysis of Y1. **(B)** GO enrichment analysis of Y2. **(C)** Heatmap of Y1 revealing the number of gene pairs interacting between two cell types. **(D)** Shell diagram of interactions between a single ligand cell and other receptor cells in Y1. Network edge thicknesses indicate ligands, and the total number of receptor pairs is indicated. **(E–H)** The top pairs with significant differences in interactions between different cells of Y1, Y2, A1 and A2. **(F)** The top pairs with significant differences in interactions between different cells of Y2. **(G)** The top pairs with significant differences in interactions between different cells of A1. **(H)** The top pairs with significant differences in interactions between different cells of A2. Fibro, fibroblasts; Glial, glial cells; Lymp, lymphocytes; Odonto, odontoblasts; Pro, proliferating cells.

Different expression profiles of the transcriptome translate into different molecular functions and biological behaviors. We compared the transcriptome expression differences of the four samples and especially focused on comparing Y1 with the other three samples. DEGs in Y1 were enriched in the ribosome and participated in translational initiation, protein localization, and cell targeting (Figure 2A). Functions related to the immune system, such as the chemokine activity and MHC class II receptor activity also played a role. These findings suggested that the formation of collagen/protein and the establishment of the immune system occurred in the pulp tissue of the pre-eruptive tooth. Y2-specific genes were involved in the organization of extracellular structure, osteoblast differentiation, and ossification, showing that cells in Y2 were further differentiated. Genes related to the molecular function of binding and adhesion in Y2 were also identified (Figure 2B; Supplementary Figure S2A). Regarding A1 and A2, DEGs contributed to cell-matrix adhesion, cell–cell junction, mitochondrial respirasome, and NADH dehydrogenase, which were related to cellular metabolic activities (Supplementary Figures S2B–E).

Cellphone DB was used to reveal intercellular communications and specific cell–cell interactions (Efremova et al., 2020). In Y1, there were frequent interactions among cell types, such as ECs, fibroblasts, odontoblasts, and MSCs (Figures 2C,D). In Y2, A1, and A2, only ECs maintained high intercellular communications with other cell types (Supplementary Figure S2F). We then constructed diagrams to reveal the specific ligand–receptor pair between different cell types (Figures 2E–H). *NOTCH* and *BMP* were found in all four samples, while the angiogenesis genes *VWF* and *VEGF*, the adhesion gene *CD34*, and the immune signals *CCL* and *CXCL* were also detected in all four samples, suggesting that these genes played a crucial role at all stages of development. *NOTCH3* was the most active receptor on the surface of MSCs, while *NOTCH1* and *4* were on ECs (Figures 2E–H). *BMP*-related signal interactions were expressed in the fibroblast of Y2 and in the odontoblasts of A1 and A2. Communication through *OSM* and *EDA* in Y1 (Figure 2E) indicated possible mineralization pathways at an early stage (Balic and Thesleff, 2015; Lin et al., 2021). ECs and glia cells interacted with other cell types through *FGF1* and *FGF2* only in Y2 (Figure 2F), suggesting that *FGF1* and *FGF2* may be more involved in apical papilla development. The expression and role of *RSPO2* in A1 and *THY1* in A2 (Figures 2G,H) indicated potential restoration pathways in mature pulp (An et al., 2018; Gong et al., 2020).

### Subpopulation characteristics of fibroblasts at multiple spatial and temporal levels

**FIGURE 3:**
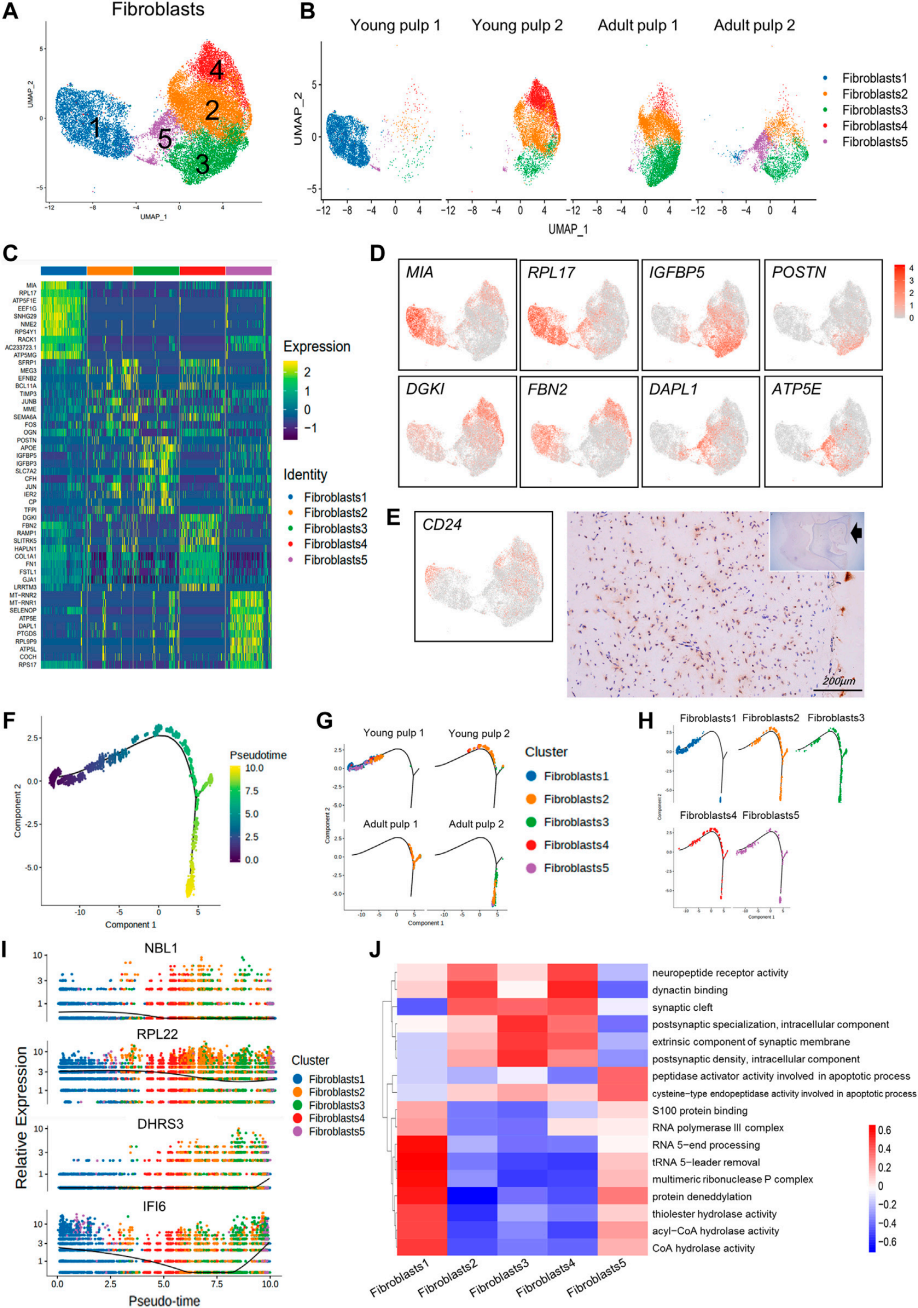
Subpopulation characteristics of fibroblasts at multiple spatial and temporal levels. **(A)** The UMAP plot of the fibroblast subpopulation. **(B)** UMAP of subgroups in the four different samples. **(C)** Heatmap of subset-specific markers. **(D)** Feature plots of marker genes in fibroblasts. **(E)** Feature plot and immunohistochemical staining of CD24 in young pulp samples. **(F)** Plot of the proposed temporal order for all cells and visualized in descending order. The temporal distribution indicates the differentiation of the pseudotemporal sequences. **(G)** The distribution of each sample in the proposed time series trajectory is plotted. **(H)** Distribution plot of each fibroblast subcluster in pseudo-sequential trajectories. **(I)** Variations in the gene expression with pseudotime. **(J)** Heatmap results of pathway enrichment for each subcluster.

Most of the dental pulp consists of fibroblasts (Nakanishi et al., 2011) and consistently represents more than half of the cell population. To reveal the cellular heterogeneity, we re-clustered the population of fibroblasts and got five subclusters (Figure 3A). Cells in Cluster 1 were from Y1, which were identified by the *MIA* gene and genes related to ribosomal proteins, such as *RPL17* and *EEF1G* (Figures 3B–D). *CD24*, considered a SCAP-specific marker, was expressed in Clusters 1, 2, and 4 of fibroblasts. Immunohistochemical staining showed that *CD24* was only localized in the dental papilla in the early stages of development (Figure 3E; Supplementary Figures S3A,B). Clusters 2 and 3 were minimal in Y1, but dominant in the other three samples, highly expressing *IGFBP5*, *EFNB2*, and *POSTN* (Figures 3B–D). These genes have been shown to be related to angiogenesis or odontogenesis of dental pulp (Du and Li, 2019; Hao et al., 2020; Yuan et al., 2019). They may play a role in the late growth of the human dental pulp. Clusters 4 and 5 represent a large proportion of Y2 and A2, respectively (Figure 3B). Cluster 4 shows a significant up-regulation of the *DGKI* and *FBN2* genes (Figures 3C, D). A death-associated protein, *DAPL1* (Figures 3C, D), ribosome, and ATP synthase genes (*MT-RNR2* and *ATP5E*) located in the mitochondria were enriched in Cluster 5, indicating that cells in Cluster 5 may have potential damage.

We predicted the temporal changes of the fibroblasts by performing a trajectory analysis (Figure 3F). The four samples were distributed in different branches on the pseudotime trajectory. Y1, present in Cluster 1, was located at the beginning of the proposed temporal sequence. The two adult samples were located at the end of the two branches (Figure 3G). However, in Clusters 2–5, there was no clear developmental sequence. (Figure 3H). Additionally, some genes also changed with pseudotime. *NBL1* expression decreased and *DHRS3* increased, while *IFI6* decreased first and subsequently increased (Figure 3I).

We used the gene set variation analysis (GSVA) to explore potential functional differences between subgroups (Hänzelmann et al., 2013). Clusters 2–4 showed a higher expression of pathways related to “synaptic” and “neuropeptide receptor activity.” Figure 3J Cluster 1, which consisted of “multimeric ribonuclease P complex” and “RNA polymerase III complex,” showed enrichment in pathways including various “hydrolase activities,” “S100 protein binding,” and “protein deneddylation” (Figure 3J). Cluster 1 might be associated with more vigorous protein synthesis and assembly functions. Cluster 5 was specifically involved in the apoptotic process through the activity of peptidase activators and cysteine-type endopeptidase activity (Figure 3J).

### Subpopulation characteristics of odontoblasts at multiple spatial and temporal levels

**FIGURE 4:**
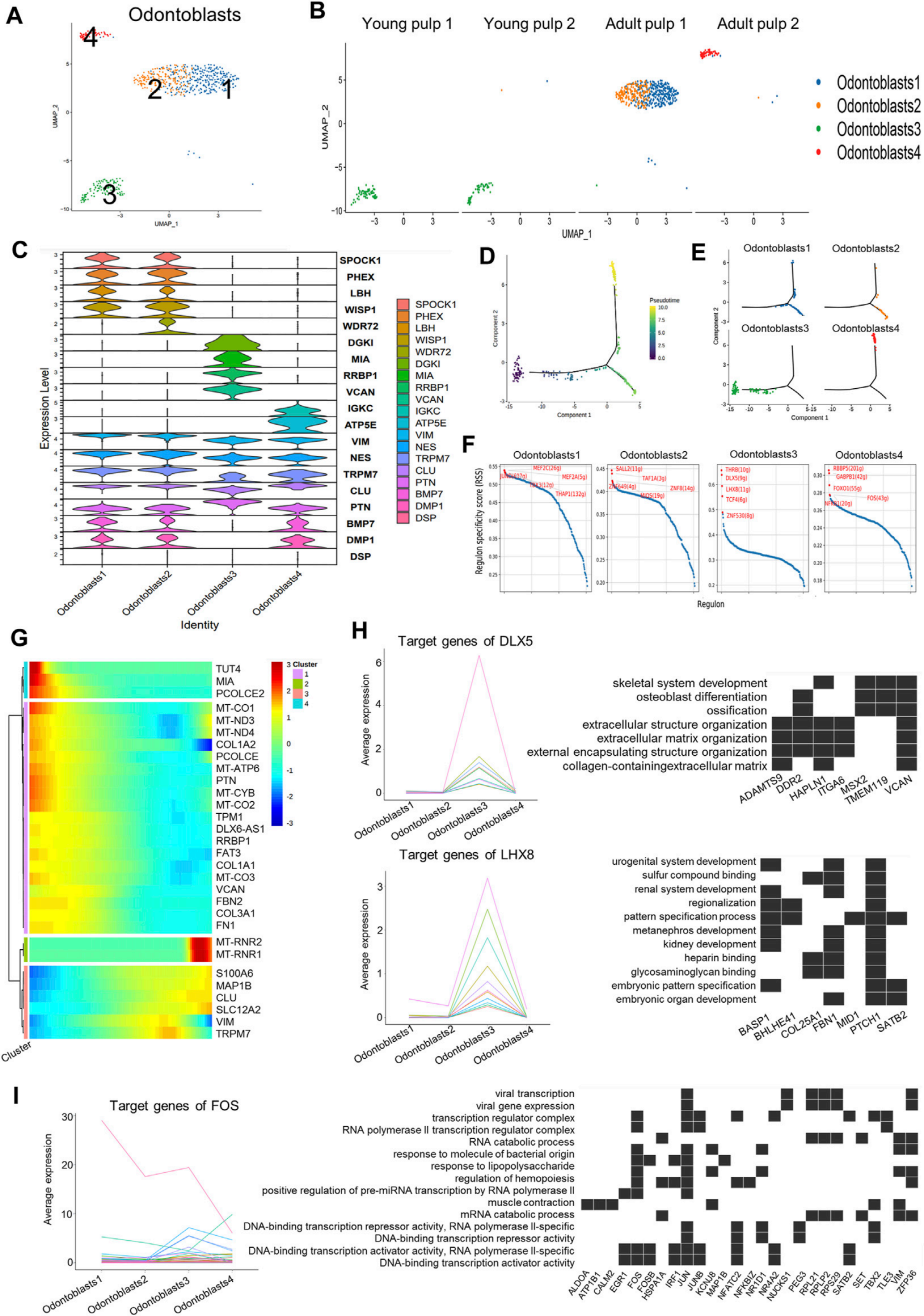
Subpopulation characteristics of odontoblasts at multiple spatial and temporal levels. **(A)** UMAP of the odontoblast subpopulation. **(B)** UMAP of the subgroups in four different samples. **(C)** Violin plot showing marker genes in odontoblasts. **(D)** The plot of the proposed temporal order for all cells, visualized in the descending order. The temporal order indicates the differentiation of the pseudotemporal sequences. **(E)** The distribution of each cluster in the proposed time series trajectory is plotted, where distinct colors indicate the cell clusters. **(F)** Scatter plots of regulon specificity for each cell type of odontoblasts, highlighting the highest top 5 regulons. **(G)** Clustered heatmap showing the dynamics of gene expression with pseudotime changes. **(H)** Left: line graph of the expression patterns of gene sets targeted by DLX5 and LHX8; right: heatmap of the relationship between target genes and the regulated pathways. **(I)** Left: line chart of the expression patterns of gene sets targeted by TFs (FOS); right: heatmap of the relationship between FOS target genes and regulated pathways.

Odontoblasts are unique cells in dental pulp that produce mineralized dentin and specifically express *DSPP* and *DMP1*. Four subclusters were found when we isolated this cell type using DEGs (Figures 4A, B). Y1 and Y2 were in Cluster 3, which highly expressed genes of *COL3A1*, *FBN2*, and *VCAN* (Figure 4C; Supplementary Figure S4A). These genes were mainly involved in dentin matrix formation (Chen et al., 2022). *PTN* with a higher expression in Cluster 3 than in the other three groups (Figure 4C) might be associated with early mineralization of dentin (Erlandsen et al., 2012). However, mineralization-related genes *DMP1* and *BMP7* were significantly decreased in Cluster 3 (Figure 4C). As for mature pulps, the odontoblasts within the two samples were not homogeneous. Clusters 1 and 2 were identified in A1, while Cluster 4 represented the major odontoblasts of A2 (Figure 4B). Cluster 2 showed minor differences compared to Cluster 1 in some mineralization-related genes, such as *WDR72* and *PHEX* (Lv et al., 2011; Katsura et al., 2014) (Figure 4C). Cluster 4 showed a higher expression of immunoglobulin kappa constant (*IGKC*) genes (Figure 4C; Supplementary Figure S4A). The appearance of Cluster 4 was similar to that of Cluster 5 in term of fibroblast composition, suggesting that odontoblasts in this cluster also undergo potential damage. However, immunohistochemical staining of *WISP1*, a marker expressing specifically in Clusters 1 and 2, showed that it did not differ significantly between samples of different time periods (Supplementary Figure S4B).

Cluster 3, as the main odontoblasts of Y1 and Y2, was positioned at the beginning of the pseudotime development trajectory (Figures 4D, E). Y1 and Y2 also showed divergence in the proposed timeline in succession. Cells in Y1 were earlier than cells in Y2 (Supplementary Figure S4C). Clusters 1, 2, and 4 were separated into two fractions at the branching point (Figures 4D, E), possibly because they were in a different physiological status or simply due to differences in the sampling. We also observed changes in the gene expression along the proposed timeline: a gradual increase in *S100A6* and *CLU* was accompanied by a decline in the expressions of *COL1A2* and *PTN* (Figure 4G).

The negative regulation pathway of the “stress fiber assembly” and the “actin filament bundle assembly” was enriched in Clusters 1 and 2, and the “endocytic vesicle lumen” and “gap junction” showed higher expression in Cluster 1 (Supplementary Figure S4D). Pathways such as “proton channel activity,” “regulation of receptor binding,” “collagen binding,” and “protein binding involved in heterotypic cell–cell adhesion” were highly expressed in Cluster 3 (Supplementary Figure S4D), indicating that young odontoblasts may be active in adhesion and interaction of various signaling pathways. The results of the path enrichment of “regulation of T cell-mediated cytotoxicity” and “angiogenesis involved in wound healing” pathways suggested that Cluster 4 of A2 could be in an injured state (Supplementary Figure S4C).

Transcription factors (TF), regulators of region-specific morphogenesis, are key orchestrators of gene activity during development. We used SCENIC to identify potential TFs that play a key role in different subgroups of odontoblast subclusters (Figure 4F). Target genes regulated by TFs were also obtained from the SCENIC analysis (Supplementary Table S4). The target genes of MEF2C, an active TF in Cluster 1, were involved in stem-cell differentiation and response to transforming growth factor beta (Supplementary Figure S4E). DLX5 and LHX8, the well-known TFs in human tooth development (Yang et al., 2019; Zhou et al., 2015), were enriched in Cluster 3 (Figure 4F). The target genes for DLX5 and LHX8 were up-regulated in Cluster 3 and were associated with the ossification and pattern-specification processes (Figure 4H). Genes regulated by FOS in Cluster 4 were associated with the response to molecules of bacterial origin, mechanical stimulus, and lipopolysaccharides (Figure 4I).

### Subpopulation characteristics of MSCs at multiple spatial and temporal levels

**FIGURE 5:**
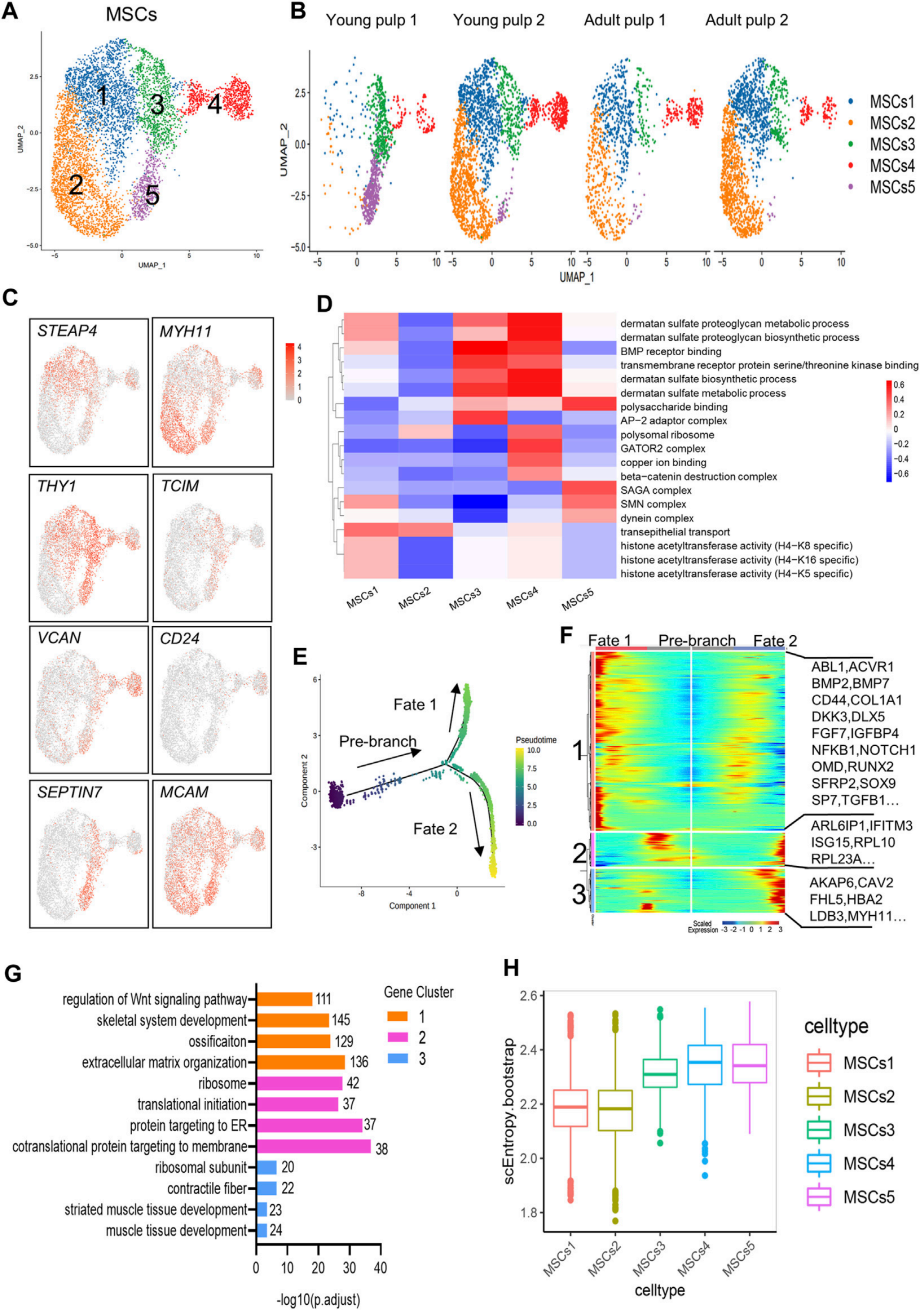
Subpopulation characteristics of MSCs at multiple spatial and temporal levels. **(A)** UMAP of the MSC subpopulation. **(B)** UMAP of subgroups in five different samples. **(C)** Feature plots of marker genes in MSCs. **(D)** Heatmap results of pathway enrichment for each subgroup. **(E)** Monocle pseudotime analysis revealing the progression of MSCs. The sequence of time indicates the differentiation of the pseudotemporal sequence. **(F)** Heatmap showing changes in the proportional of differently expressed genes in three branches, divided into three major gene clusters (labels on the left). Right: specific representative genes in each gene cluster. **(G)** GO analysis of differently expressed genes associated with three gene clusters. **(H)** The box line plot of the entropy distribution of MSC subpopulations.

MSCs with multiple differentiation potential and high regenerative capacity are considered relevant for organ tissue development and repair *in vivo* (Yu and Klein, 2020). MCAM/CD146, THY1/CD90, and CD24 are generally considered markers of dental mesenchymal stem cells (Gronthos et al., 2000; Miura et al., 2003; Sonoyama et al., 2008), and are located in the perivascular niche and apical papilla of human dental pulps (Figure 3E; Supplementary Figure S5A, B). Herein, the MSCs of the four samples were subdivided into five groups (Figures 5A, B). The distribution of Clusters 1 and 2 was consistent with that of Y2, A1, and A2, but very few were detected in Y1. The two clusters specifically expressed genes related to ion regulation and cytoskeleton formation, including STEAP4, ABCC9, MYH11, and NET1 (Figure 5C; Supplementary Figure S5C). However, THY1/CD90 expression was significantly negatively regulated in Cluster 2. A high expression of THY1/CD90 was found in Cluster 3 and represented a large proportion of Y1 (Figures 5B,C). Furthermore, Cluster 3 surprisingly expressed the genes related to lipid synthesis and transport, APOE and CYP1B1. Furthermore, the WNT regulator TCIM and the MMP regulator TIMP1 were highly expressed in this cluster (Figure 5C; Supplementary Figure S5C). Cluster 4 explicitly expressed small leucine-rich proteoglycans such as LUM and VCAN, which were similar to the expression profile of odontoblasts (Figures 4C, 5C; Supplementary Figure S5C). Therefore, Clusters 3 and 4 may be prerequisite MSC populations for odontoblasts. A specific marker of SCAP, CD24, is presented in Cluster 4 (Figure 5C). Cluster 5 mainly existed in Y1, distinguished by the particular expression of genes of the SEPTIN family (especially SEPTIN7 and SEPTIN4), which may be associated with proliferation or cytokine apoptosis (Figure 5C; Supplementary Figure S5C). Another marker gene for oral MSCs, MCAM/CD146, was found in all subpopulations.

In the pathway-level differential analysis, more information on the cell subpopulation was found. Cluster 1 was related to the function of histone acetyltransferase activity. The expression of “transepithelial transport” was high in Clusters 1 and 2. Cluster 3 and 4 were enriched with BMP receptor binding, suggesting that they were associated with odontoblast differentiation and mineralization of dentin. The biosynthetic and metabolic pathways of dermatan sulfate were also actively reflected in Clusters 3 and 4. However, these two subgroups showed variations in the expression of the “AP-2 adaptor complex” and “beta-catenin destruction complex.” In Cluster 5, “SAGA complex,” “dynein complex,” and “polysaccharide binding” were significantly expressed and could contribute to cell division and transcription (Figure 5D).

There were no significant differences in the distribution of Clusters 1 to 4 on the temporal trajectory (Supplementary Figure S5D), and differentiation and development relationships between these subgroups could not be inferred based on the monocle trajectory analysis. The gene expression patterns involved in the continuum transition were further analyzed (Figures 5E,F). GO terms “regulation of Wnt signaling pathways,” “ossification,” and pathways involved in “extracellular matrix organization” (*BMP7*, *OMD*, *RUNX2*, and *SP7*) that predict dentinogenesis or osteogenesis were enriched in Cluster 1, and their expressions decreased with the Fate 2 branch (Figures 5F,G). Genes in Clusters 2 and 3 were related to “protein targeting to the ER” and “muscle tissue development” (Figure 5G), suggesting that two different directions (mineralization and myogenesis) appeared during MSC differentiation.

SLICE analysis was used to explore the genealogy and differentiation status within subpopulations of MSCs by calculating the entropy (Guo et al., 2017). Clusters 3–5 showed higher entropy values, indicating higher differentiation potential (Figure 5H).

**FIGURE 6:**
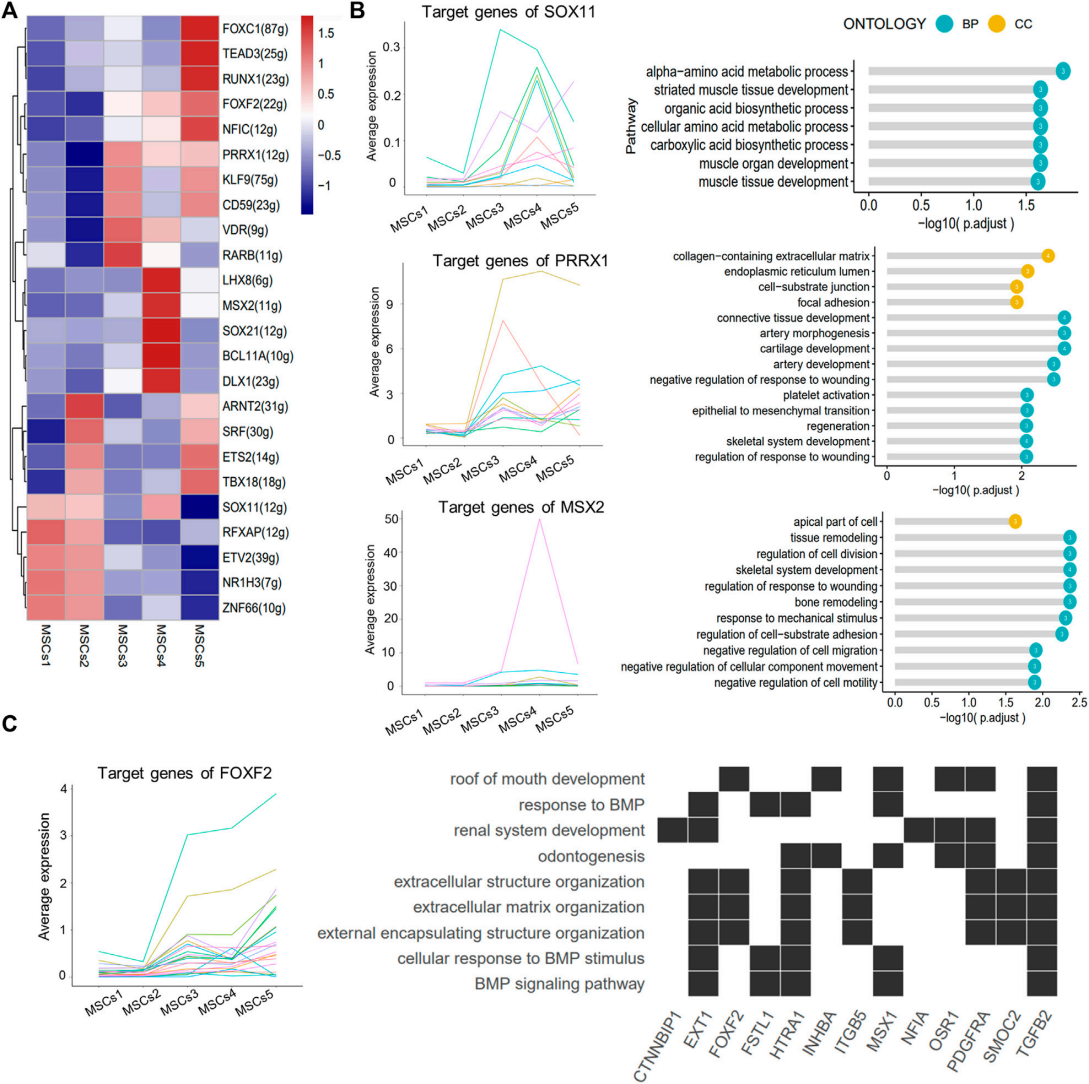
SCENIC-identified major regulons in MSCs. **(A)** Heatmap showing the top 5 area under curve (AUC) clusters of regulons in each cell type. **(B)** Left: line graph showing the expression patterns of gene sets targeted by TFs (SOX11, PRRX1, and MSX2); right: lollipop graph showing the target gene pathways regulated of key transcription factors (SOX11, PRRX1, and MSX2). Longer lollipop bars represent more significant enrichment results, and the values inside the circles represent the number of genes enriched for that pathway. **(C)** Line graph of the expression patterns of the gene sets targeted by TF FOXF2 (left); heatmap of the relationship between the FOXF2 target genes and the regulated pathways (right).

SCENIC identified major regulons in each group (Figure 6A; Supplementary Table S5). Except for TFs in Clusters 4 and 5, all other TFs served in multiple subgroups at the same time. The target genes regulated by the SOX11, PRRX1, and MSX2 TFs were enriched in the “striate muscle tissue development,” “focal adhesion,” and “negative regulation of cell migration or motility” pathways (Figure 6B). The downstream genes of the key TF FOXF2 in Cluster 5 were clearly involved in the pathways associated with “odontogenesis” and the “BMP signaling pathway” (Figure 6C).

## Discussion

In this study, we combined sequencing sample data from one primary sample with published data of three samples extracted from a public database (Krivanek et al., 2020; Pagella et al., 2021b) to explore the gene expression characteristics of human dental pulp cells at different spatial and temporal levels.

Pulp cells at different stages of development showed variations in the proportion of cell types. MPs, lymphocytes, and glial cells showed variability in samples at different timepoints of development. Some variations in the number of endothelial cells and glial cells may be associated with the developmental processes of the dental pulp. Previous studies have shown that endothelial cells appear in the bell stage and gradually increase with vascular and tooth development (Takahashi et al., 2012; Shadad et al., 2019). Neural axons begin to enter the apical papilla progressively only after crown formation, although early innervation was present (Fristad et al., 1994). It is interesting that MPs are significantly more prevalent in Y1 than in the other three samples. *CD163*, a marker gene for macrophages of the M2 phenotype, was differentially expressed in Y1 compared to the other three samples and its protein was found in the pulp crown in the early stage of development (Figure 1E). The M2 phenotype macrophages co-localize with Schwann cells in the human dental pulp and play a role in promoting odontogenic differentiation of human dental pulp cells (Park et al., 2017; Yoshiba et al., 2020). The large proportion of dendritic cells in Y1 MPs (Supplementary Figure S1B) is consistent with previous studies describing their possible role in dentin formation (Quispe-Salcedo and Ohshima, 2021). The interaction of *OSM/OSMR* between MPs and ECs in Y1 could be related to its role in the early sample (Figure 2E). A study exploring the immune microenvironment of the mouse mandible has revealed that a population of MPs overexpressed the *OSM* gene while in traffic with MSCs and promoted osteogenesis through this pathway (Lin et al., 2021). All this information suggests that MPs and related genes could play a role in the development of teeth; therefore, there were more MPs present in young teeth.

We identified the characteristics of human odontoblast expressions and potential mechanisms for transcriptional regulation during development. Odontoblasts can develop, differentiate, and secrete predentin–dentin components according to specific spatiotemporal patterns (Balic and Thesleff, 2015). Due to the non-renewable nature of human teeth and ethical restrictions, many of the current research findings on animal experiments cannot be extended to the human domain. Through this high-throughput sequencing study, we determined that odontoblasts highly expressed ion channel-related genes (*TRPM7*, *SLC12A2*, and *SPOCK3*) and neural-related genes (*ST8SIA1* and *MAP1B*) (Figure 1F; Supplementary Figure S1C), where the role of *TRPM7* and *MAP1B* in odontoblast differentiation has been demonstrated (Cui et al., 2014; Maurin et al., 2009). Ion channels regulate and preserve calcium and pH homeostases, which are essential for proper biomineralization of the enamel and dentin, and mutations in related genes often lead to catastrophic changes in tooth development (Duan, 2014). In this study, a total of four DEG-based odontoblast subgroups were found, and two differentiation directions were identified by pseudotime analysis (Figures 4A,C). Cluster 3, located in immature young pulps, as an earlier subpopulation in the dentin-forming phase, could have a more vigorous secretory function. *PTN* was expressed in all odontoblasts, but at a higher level in Cluster 3 (Figure 4C). Previous research has shown that *PTN* was expressed in odontoblasts and in the basement membrane of organs that undergo epithelial–mesenchymal interactions (Erlandsen et al., 2012). The intensity of the *NES* expression increased with the progression of odontoblast differentiation, which was consistent with previous studies (Balzano et al., 2021; Quispe-Salcedo et al., 2012). We also identified genes that may be marker genes for early odontoblasts, such as *DGKI* and *RRBP1*. Some genes were highly expressed in mature subpopulations but not in developmental subpopulations, such as *PHEX* and *WDR72* (Figure 4C), which were associated with defective diseases (Katsura et al., 2014; Lv et al., 2011), possibly suggesting that they played a more significant role in maintaining homeostasis than promoting development. *WISP1* was highly expressed in Clusters 1 and 2. However, immunohistochemical staining did not differ significantly between samples (Supplementary Figure S4B). This could be due to the deviation of the transcriptome-sequencing results from the final protein expression, or it could be related to the long developmental cycle of human teeth compared to mice.

We identified a population of earlier MSCs within a large heterogeneous stem cell population and found that dental pulp stem cells can differentiate in two directions: mineralization and myogenesis. In the past, we used *MCAM/CD146*, *THY1/CD90*, and *CD24* as markers of dental MSCs (Gronthos et al., 2000; Miura et al., 2003; Sonoyama et al., 2008), but their expression levels vary within stem cell populations (Figure 5C). As the pre-eruptive sample with the earliest stage of development of the four samples, Y1 contained Clusters 3–5 with a high expression of *THY1* (Figure 5C). Therefore, *THY1* can be used as a marker for the early stage of human dental pulp stem cells. One study has shown that *THY1* was present in continuously growing mouse incisors with a dramatic decrease when growth rate homeostasis is established (An et al., 2018). Cluster 5 showed an up-regulation of *SEPTIN*, which was significantly decreased in other samples (Figure 5C). SEPTIN, a cytoskeletal protein, is important for development and differentiation (Chen et al., 2021; Woods and Gladfelter, 2021). Thus, Cluster 5 might represent a progenitor cell population of dental pulp stem cells, with stronger proliferation and differentiation potential. Combining gene expression, temporal trajectory, TF, and GO analysis, we hypothesized that dental pulp stem cells exhibit two different differentiation trends. The first, Cluster 4 differentiates toward mineralization, while Clusters 1 and 2 differentiate toward myogenesis. The MSC niche is believed to be associated with the vasculature throughout the body in the form of pericytes (Wong et al., 2015). Pericytes are closely related to vascular smooth muscle cells (VSM) (Armulik et al., 2005). Thus, we hypothesize that in addition to differentiating toward odontoblasts, human pulp stem cells also differentiate into a population such as pericytes that reside in mature pulp for future restoration and defense.

There were some interesting differences and similarities between the two young samples and between the two mature samples. Temporally, Y1 was 2 years younger than Y2, with a shorter root. As reflected in their DEGs: Y1 was mainly enriched in the ribosome and participated in translational initiation, protein localization, and targeting, while Y2 was more involved in the organization of extracellular structure, osteoblast differentiation, and ossification (Figures 2A, B). In pseudotime trajectory, Y1 was always at an earlier stage than Y2 (Figures 3G, 4E). Previous studies found that mineralization-related genes were more frequently expressed at the mature pulp of the tooth (Kim et al., 2016; Joo et al., 2018). Spatially, Y1 contained mature pulp and apical papilla, while Y2 only contained the apical part. We had expected they were homogeneous in the UMAP, but as it turned out, they showed big differences in cell distribution (Figures 3B, 5B), even though they shared some similarity in the odontoblast cluster (Figure 4B). A1 and A2 were similar in the composition of MSCs subpopulations (Figure 5B). But we identified fibroblasts and odontoblasts in A2 with the characteristic expression of apoptosis and inflammatory responses genes (Figures 3B, 4B). However, these samples were extracted from healthy mature teeth. There are several reasons which may cause inconsistency between the 2 adult samples: individual differences and batch effect. It is also possible that surgery or sampling has caused a shift in cell status. We also propose a hypothesis based on the results of our analysis: A2 might be in a state of potential inflammatory response prior to clinical symptoms. Therefore, more samples should be included in subsequent studies to rule out this problem.

For the first time, our study compared healthy pulpal characteristics at different time points and sites, and the characteristics of fibroblasts, odontoblasts, and MSCs were analyzed in depth. This study provides new information on the development of dental pulp and pulp stem cells through high-throughput sequencing methods. The previous fragmented information was summarized and validated by single-cell sequencing. These findings could provide promising new directions or goals for pulp regeneration. But our sample size was small and was affected by individual differences. Therefore, subsequent studies should obtain more comprehensive and continuous analyses by expanding the sample size and establishing a complete temporal developmental timeline. Basic experiments and targeted analyses need to be conducted in conjunction with multiomics technologies to reveal the mechanisms of pulp development and disease.

## Data Availability

The datasets presented in this study can be found in online repositories. The names of the repository/repositories and accession number(s) can be found below: https://www.ncbi.nlm.nih.gov/, GSE202476.
