# Erratum: Atlas of human dental pulp cells at multiple spatial and temporal levels based on single-cell sequencing analysis

**DOI:** 10.3389/fphys.2023.1331650

**Published:** 2023-11-09

**Authors:** Frontiers Production Office

**Affiliations:** Frontiers Media SA, Lausanne, Switzerland

**Keywords:** tooth development, single-cell RNA sequencing, odontoblasts, dental stem cells, pulp regeneration

Due to a production error, Figure 1, 4, and 5 were erroneously cropped in the PDF version of the published article.

The publisher apologizes for this mistake. The original version of this article has been updated.

