# Supplementary material for: Atlas of human dental pulp cells at multiple spatial and temporal levels based on single-cell sequencing analysis
Source: Front Physiol. 2022 Oct 4;13:993478. doi: 10.3389/fphys.2022.993478 (PMC9578252; doi:10.3389/fphys.2022.993478)
Supplement: Supplementary file 1 [file Table1.DOCX]

# Supplementary Material

**
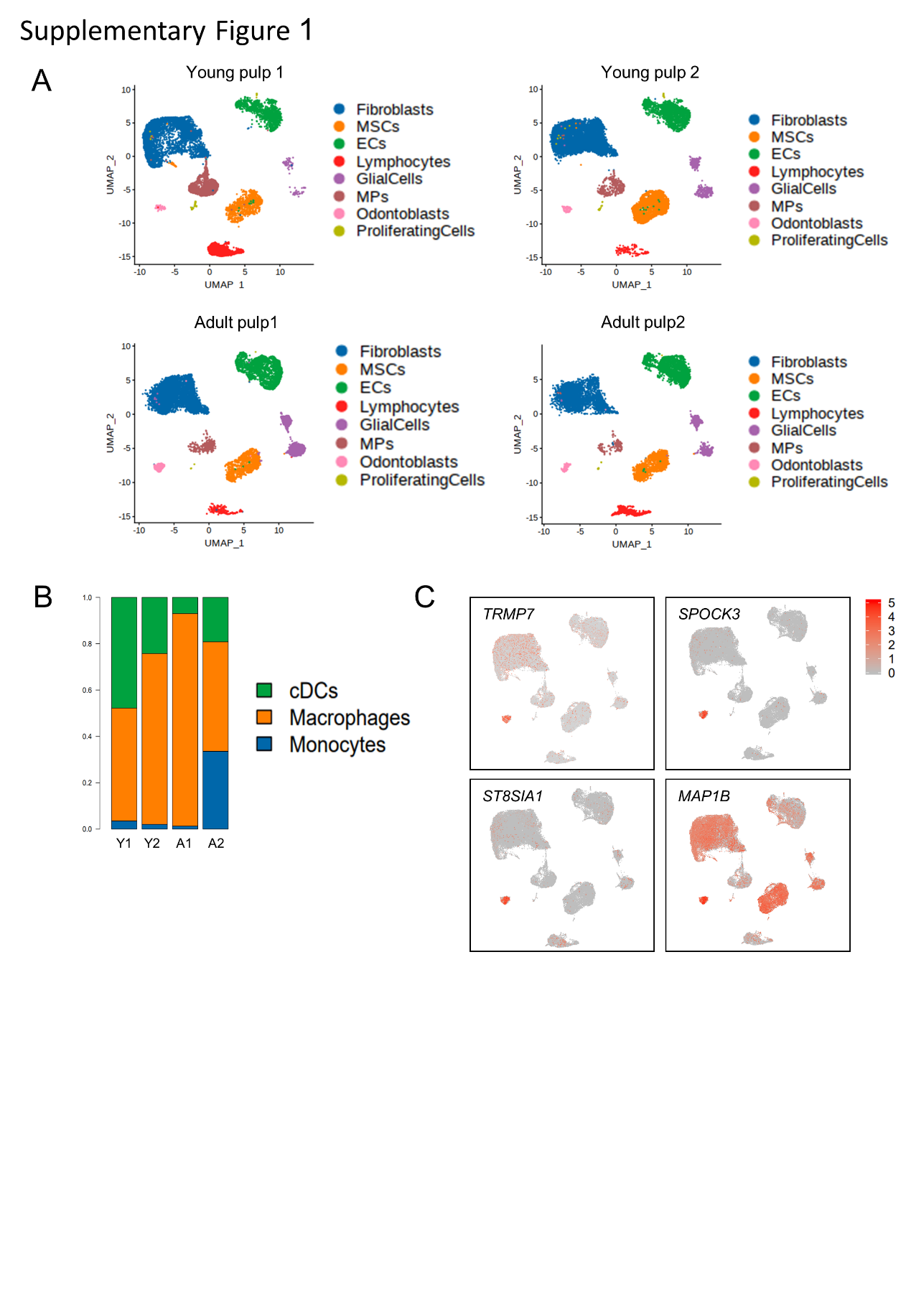
**

**Supplementary Figure 1.** A) Human pulp cell clusters identified in four separate samples. B) Relative proportion of different MPs in four pulp tissues. cDCs: conventional dendritic cells. C) Feature plots of genes in odontoblasts.


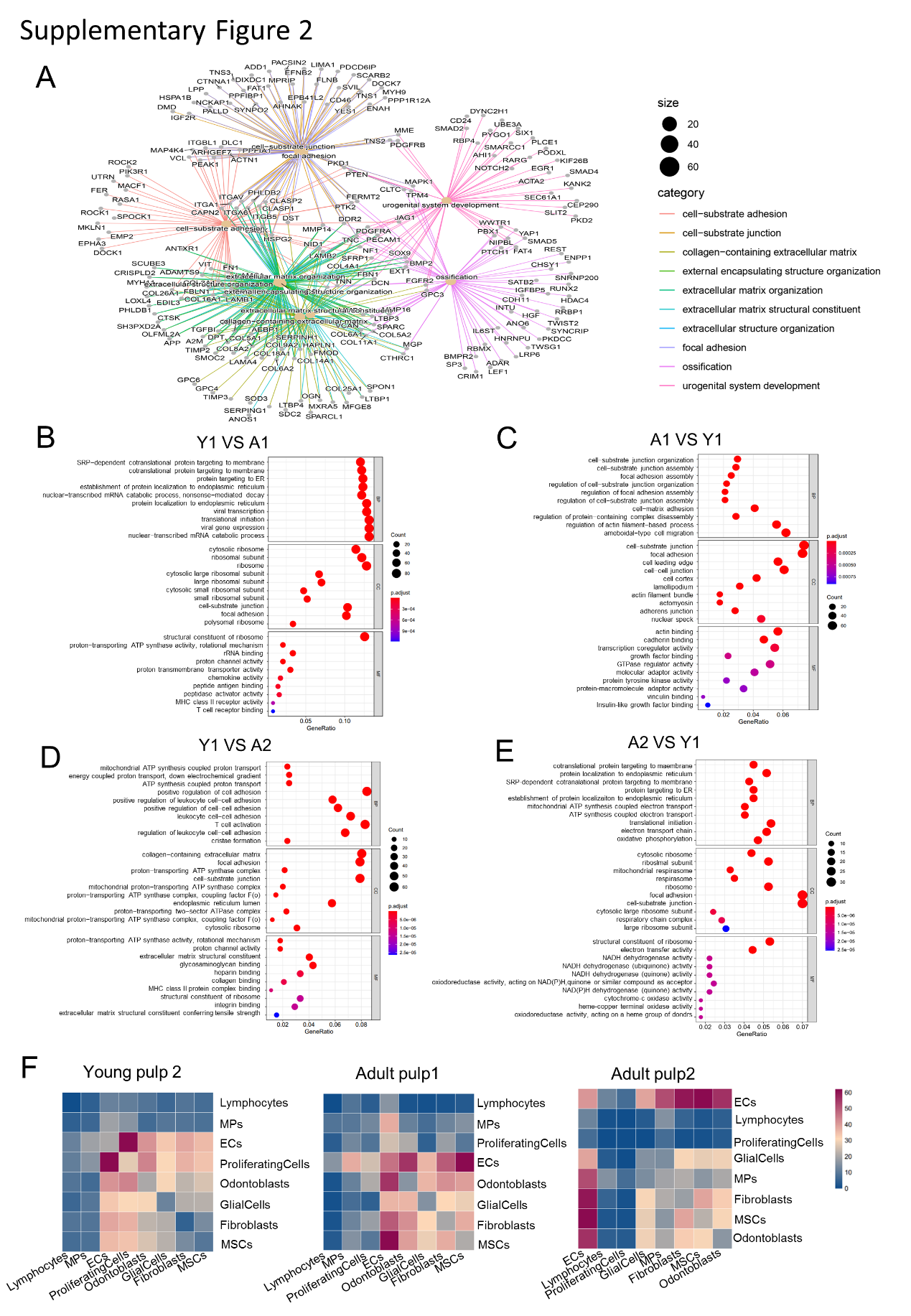


**Supplementary Figure 2.** A) Netplot diagram illustrating the relationship between genes and pathways in Yong pulp 2. B) Dotplot of the gene ontology pathway of Y1 VS A1. C) Dotplot of the gene ontology pathway of A1 VS Y1.D) Dotplot of the gene ontology pathway of Y1 VS A2. E) Dotplot of the gene ontology pathway of A2 VS Y1. F) Heatmaps of three samples showing the number of pairs of interactions between two cell types in different samples.


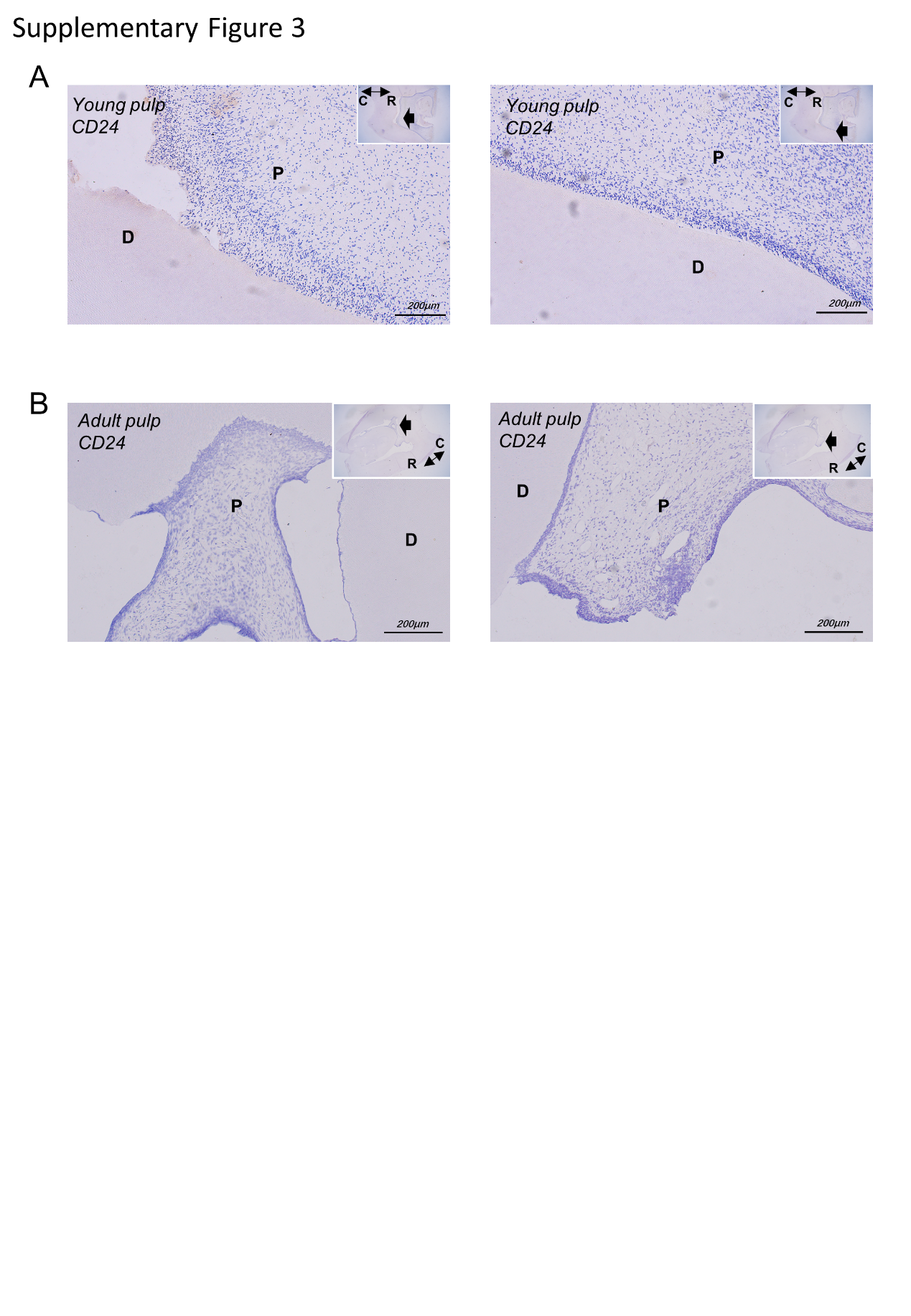


**Supplementary Figure 3.** A) Immunohistochemical stainings of CD24 in crown of young pulp. B) Immunohistochemical stainings of CD24 in adult pulp. P: Pulp; D: Dentin.


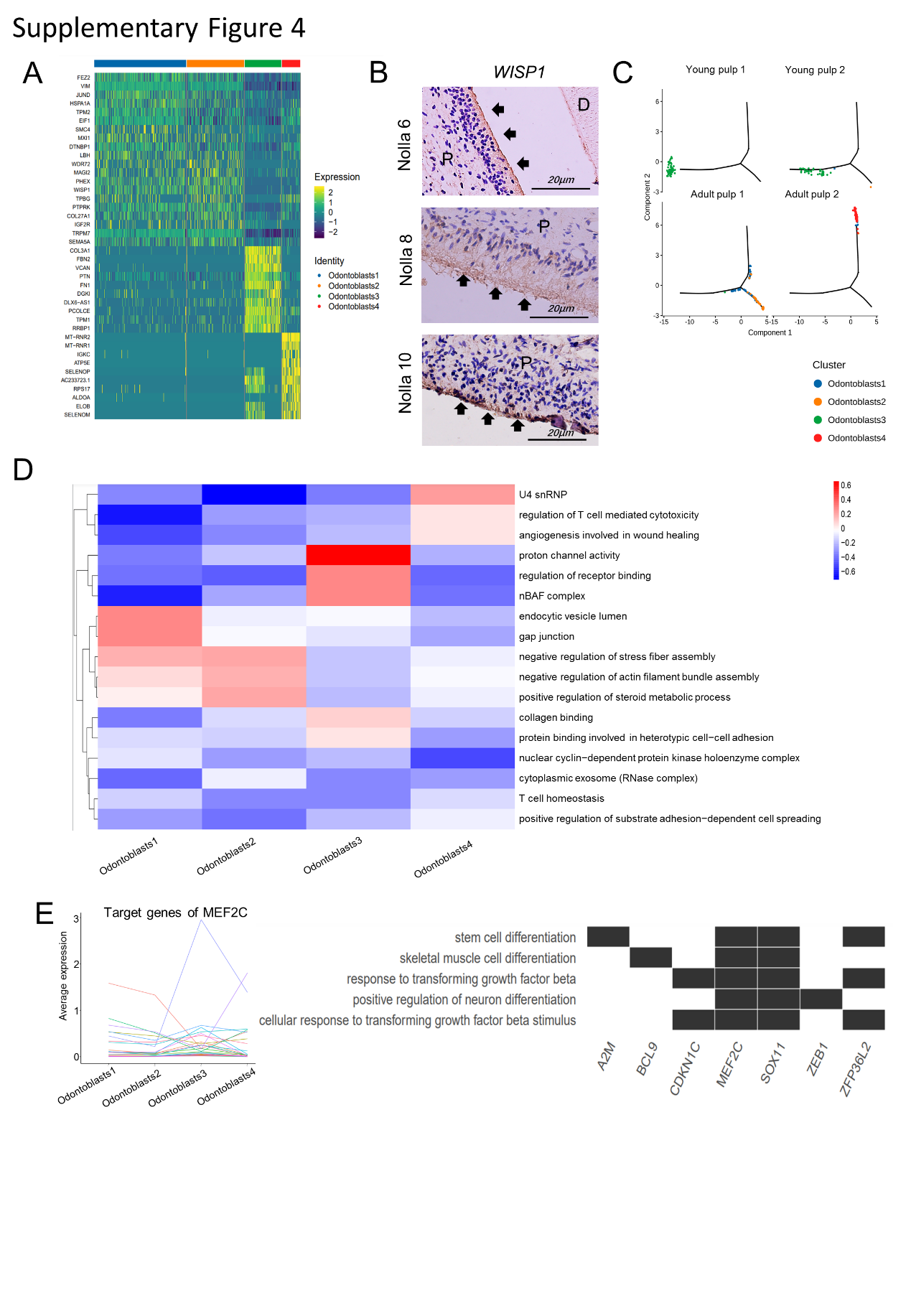


**Supplementary Figure 4**. A) Heatmap of subset-specific markers. B) Representative images of WISP1 immunohistochemical staining in odontoblasts (black arrows) at Nolla 6, 8 and 10. P: Pulp. D: Dentin. C) The distribution of each sample in the proposed time series trajectory is plotted, where distinct colors indicating the cell types in each sample. D) Heatmap results of pathway enrichment for each odontoblast subgroup. E) Left: line graph of expression patterns of TF-targeted gene sets (MEF2C); right: heat graph of the relationship between target genes of key transcription factors (MEF2C) and regulated pathways.


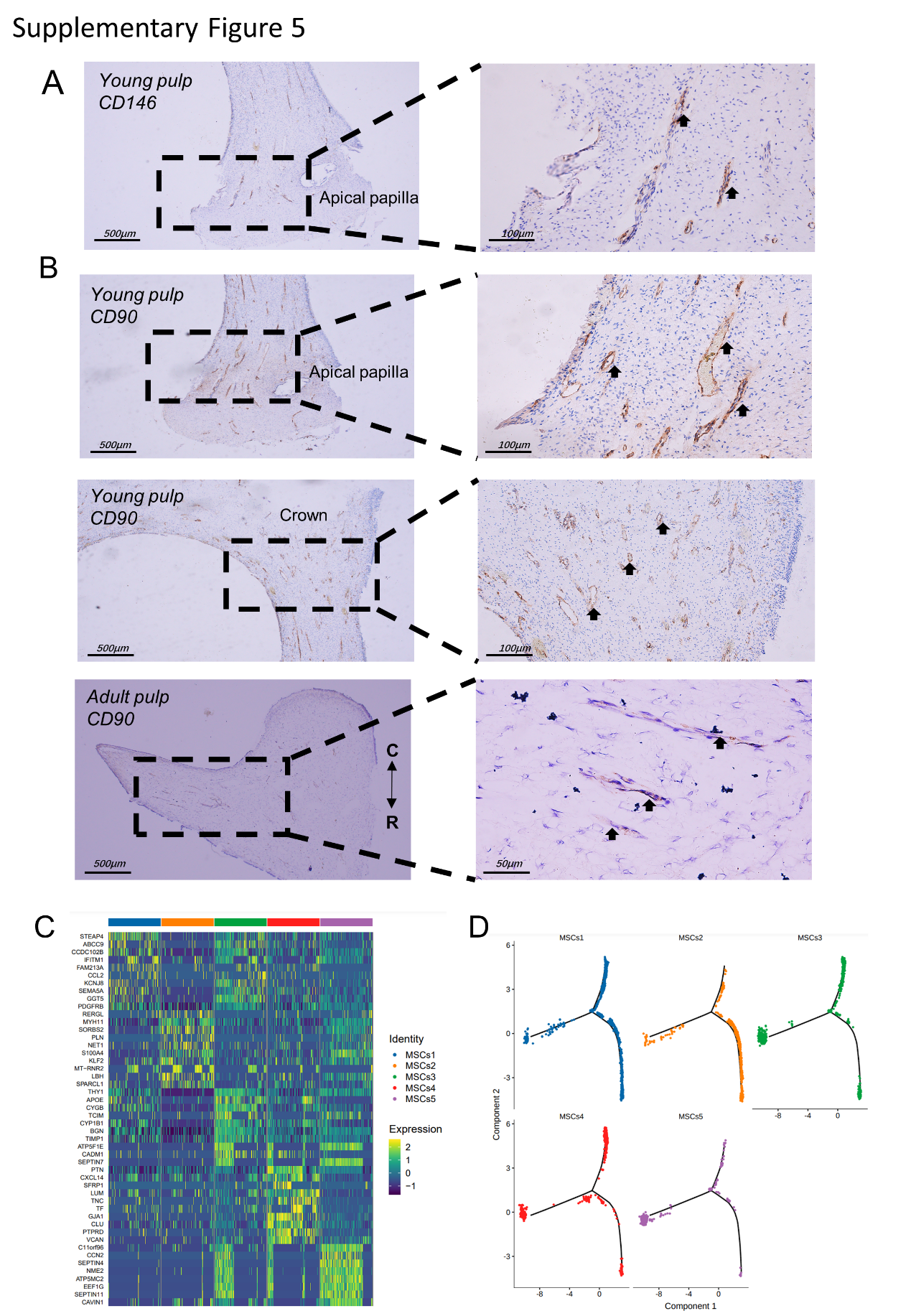


**Supplementary Figure 5**. A) Immunolocalizations of the CD146 antigen on blood vessels (black arrows) in human dental pulp. B) Immunolocalizations of the CD90 antigen (black arrows) in human dental pulps. C) Heatmap of subset-specific markers. D) Distribution plot of each MSCs type individually in pseudo-sequential trajectories.

**Supplementary Table 1:** Samples information

| Sample tag Sources Sex Age Characteristics Platform(Sequencing depth) |
| --- |
| Y1 Clinical male 13 Pulp of a third molar with less than 10x Chromium  Collection 1/3 root completed (pre-eruptive) (50k reads/cell)  Y2 GSM4365609 female 15 Apical papilla of a third molar with 10x Chromium  2/3 root completed pulp (50k reads/cell)  A1 GSM4365610 female 24 Adult mature pulp of a third molar 10x Chromium  (50k reads/cell)  A2 GSM4998458 Unknown 18-35 Adult mature pulp of a third molar 10x Chromium  (50k reads/cell) |

**Supplementary Table 2:** Annotated genes of dental pulp cell types

| Cell type Markers |
| --- |
| Fibroblasts LUM,DCN,COL1A1  Mesenchymal stem cells THY1,ACTA2,NOTCH3,MYH11,FRZB  Endothelial cells CDH5,PECAM1,VWF,EMCN  Proliferating Cells TOP2A,MKI67,TUBA1B,TYMS  Lymphocytes CD2,CD3D,TRAC,TRBC2,CD79A,NKG7,KLRD1  Glial Cells PLP1,SOX10,COL28A1,SCN7A,GJC3,MBP,MPZ  Odontoblasts TRPM7,DMP1,S100A13  Mononuclear phagocytes LYZ,C1QA,MRC1,CD68,CD163,APOE, CSF1R, FCGR3A,  Monocytes LYZ,CD14,FCN1,VCAN  Macrophages LYZ,CCL3,CCL4,IL1B  Conventional dendritic cells CD1C,CD1E,FCER1A,LAMP3,CCR7,XCR1,CLEC9A |

**Supplementary Table 3:** Number of different cell types in each sample.

| Y1 Y2 A1 A2 Total |
| --- |
| Fibroblasts 5663 7092 7086 3456 23297  Mesenchymal stem cells 1061 2433 1056 1849 6399  Endothelial cells 1398 1693 2091 2512 7694  Proliferating Cells 71 67 4 21 163  Lymphocytes 1661 119 162 568 2510  Glial Cell 93 376 1759 643 2871  Mononuclear phagocytes 1849 381 304 163 2697  Odontoblasts 66 84 585 62 797  Total 11862 12245 13047 9274 46428 |

**Supplementary Table 4.** Gene sets targeted by each individual TF in Odontoblasts.

**Supplementary Table 5.** Gene sets targeted by each individual TF in MSC.
