# Supplementary figures and images for: Atlas of human dental pulp cells at multiple spatial and temporal levels based on single-cell sequencing analysis
Source: Front Physiol. 2022 Oct 4;13:993478. doi: 10.3389/fphys.2022.993478 (PMC9578252; doi:10.3389/fphys.2022.993478)

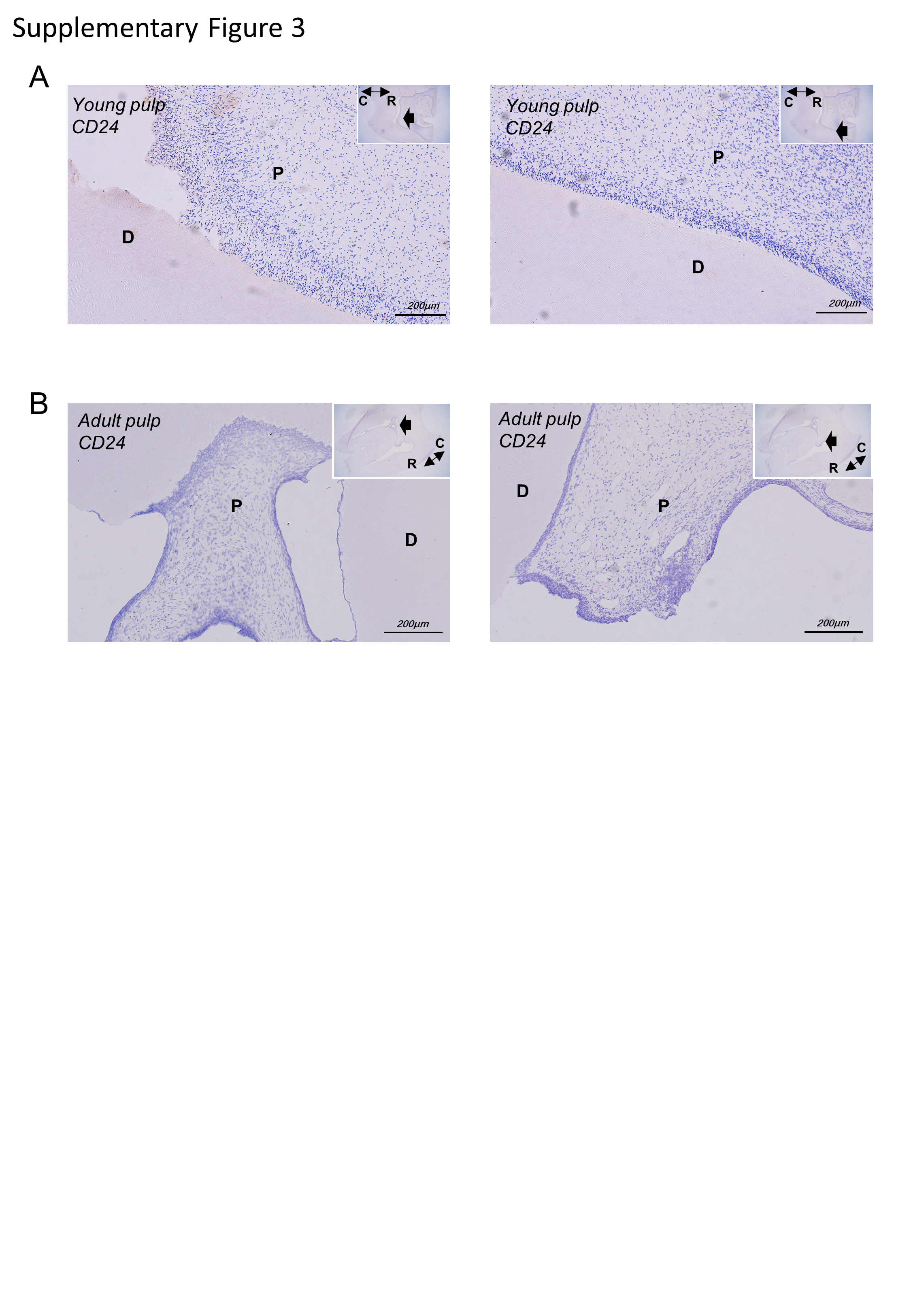

Supplement: Supplementary file 2 [file Image3.TIF]

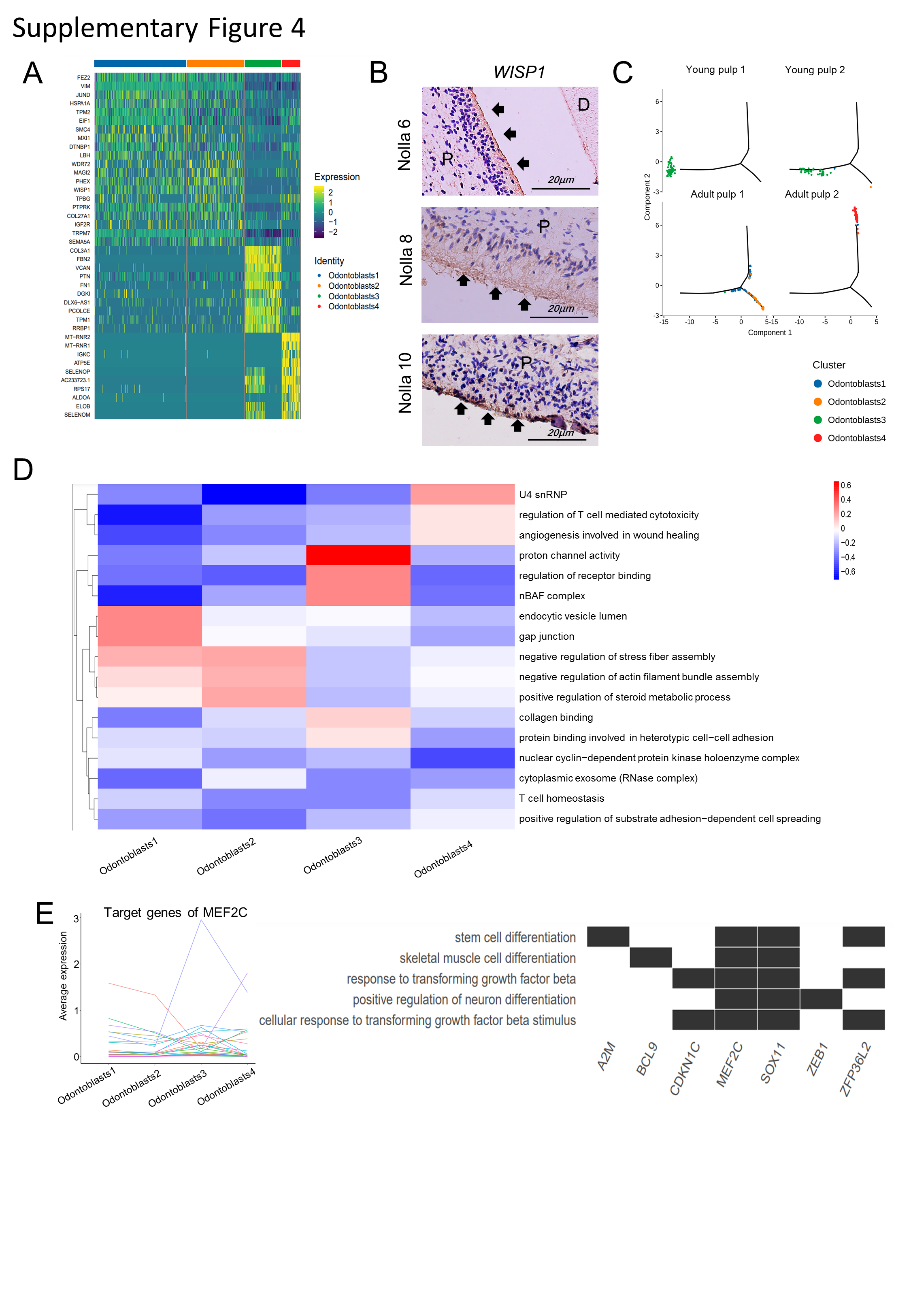

Supplement: Supplementary file 3 [file Image4.TIF]

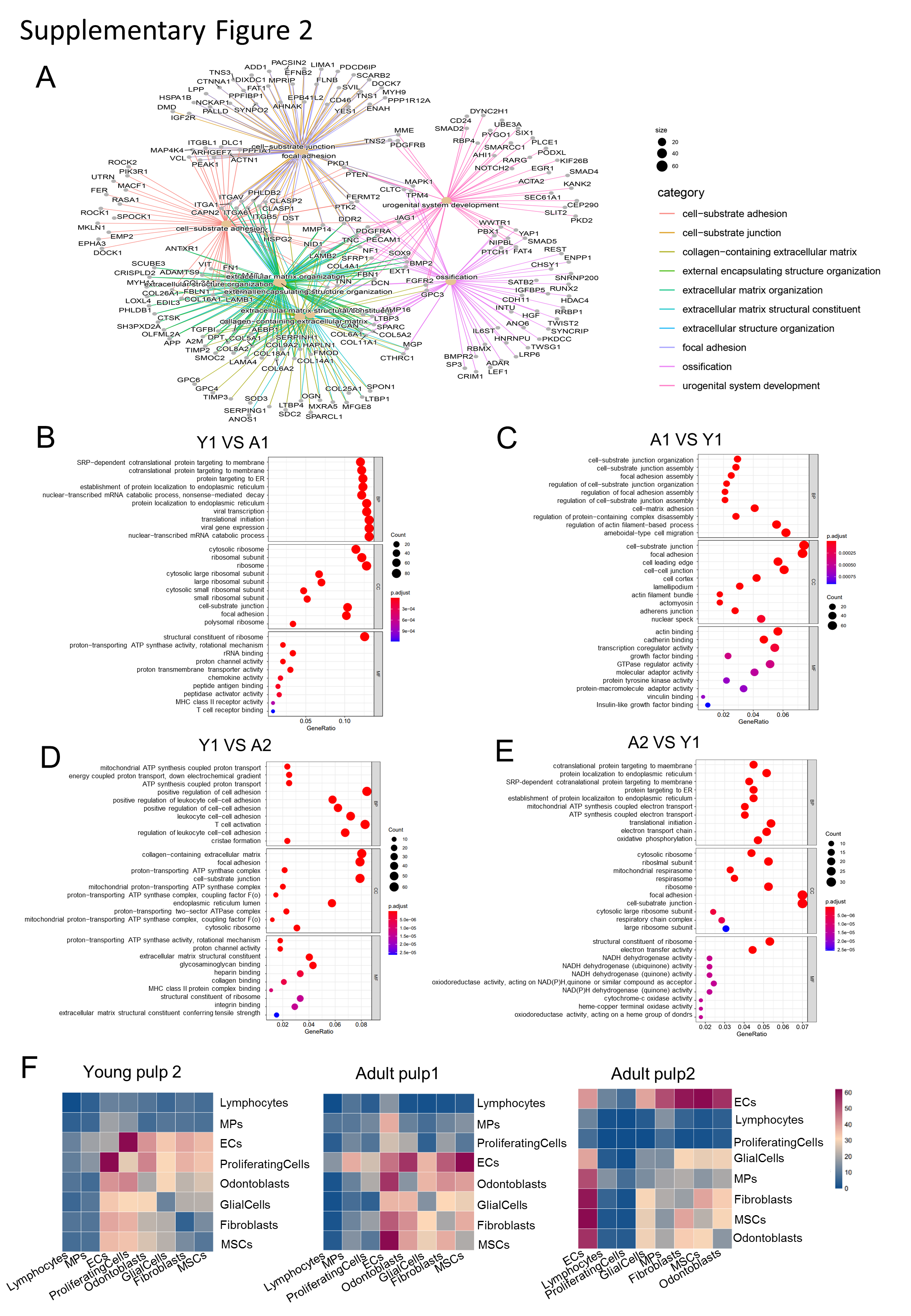

Supplement: Supplementary file 4 [file Image2.tif]

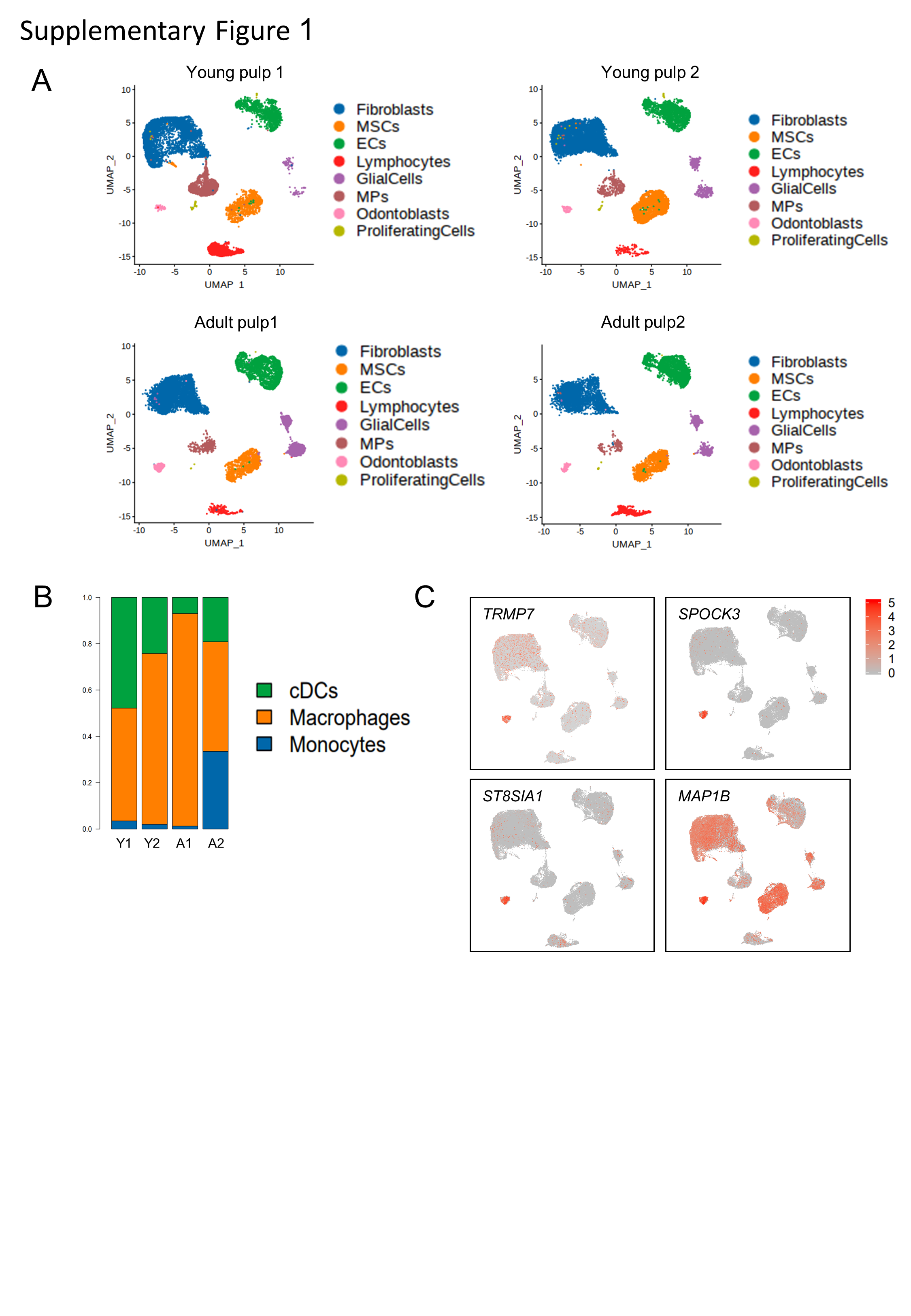

Supplement: Supplementary file 5 [file Image1.TIF]

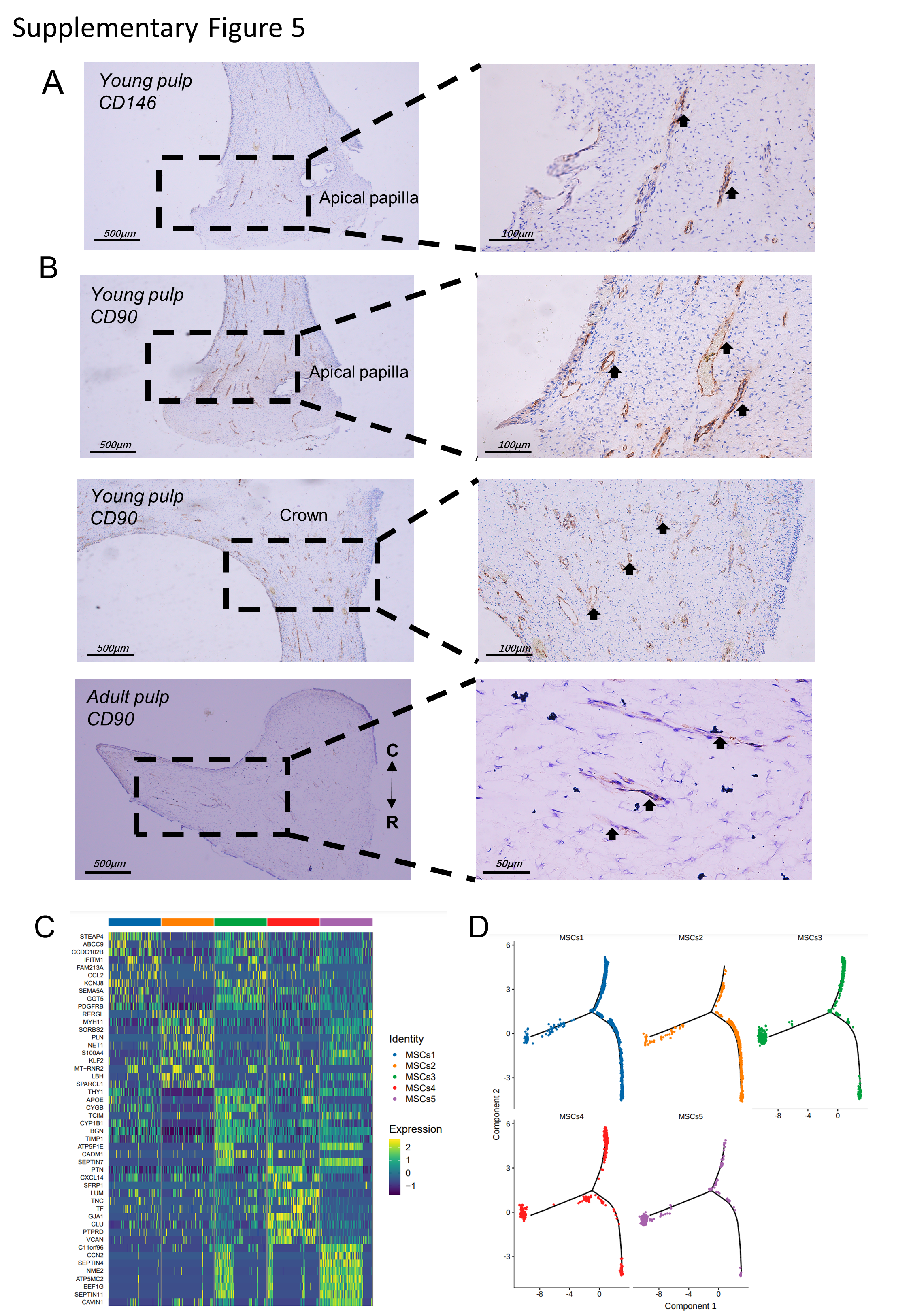

Supplement: Supplementary file 6 [file Image5.TIF]
